# FedAD: adaptive federated disentanglement for domain generalization in unlabeled medical imaging

**DOI:** 10.3389/fradi.2026.1829701

**Published:** 2026-07-29

**Authors:** Swetha Kodhandaraman, Hemalatha K, Anandita Prabhakar, Shreyas Kempapuram

**Affiliations:** School of Computer Science and Engineering, Vellore Institute of Technology, Chennai, India

**Keywords:** adaptive optimization, disentangled representation, domain generalization, federated learning, medical imaging, self-supervised learning

## Abstract

Three barriers significantly hinder the use of deep learning in medical imaging: poor generalization to new clinical domain shifts, label scarcity, and data privacy. A unified framework that learns from unlabeled, decentralized data while optimizing for generalization is desperately needed, even if Federated Learning (FL), Self-Supervised Learning (SSL), and Domain Generalization (DG) provide partial solutions that often operate under contradictory assumptions. We present FedAD: Adaptive Federated Disentanglement, a unique framework that uses two key ideas to handle these problems in a synergistic way. First, a federated semantic disentanglement objective (FedSD) explicitly distinguishes between the domain-invariant semantic characteristics and domain-specific variants using a non-adversarial orthogonality constraint. Second, in order to prevent premature convergence and enhance resilience, an adaptive teacher-student alignment (ATSA) curriculum dynamically modifies the generalization pressure based on the stability of the global model. This dual technique creates a strong feature encoder by forcing the model to learn what it sees as opposed to where it sees it. FedAD outperforms current approaches in terms of generalization to unseen target domains, as demonstrated by its validation on publicly available medical datasets. Our strategy concurrently addresses privacy, label scarcity, and domain change, paving the road for useful, reliable, and fair medical AI.

## 1 Introduction

The significant potential of deep learning in the field of medical imaging is profoundly obstructed by a trio of interrelated challenges that create a substantial barrier to the translation of research into practical clinical applications. Firstly, stringent regulations regarding data privacy result in unavoidable data partitions, which restrict models to smaller, less diverse datasets, potentially leading to algorithms that are biased and ineffective (1). Secondly, the considerable expense and time required for expert labeling result in a significant annotation bottleneck, which impedes the scalability of artificial intelligence solutions across the extensive array of diseases and imaging modalities. Lastly, when models are deployed in new clinical settings, they often struggle to generalize due to domain shift (2). This represents a critical issue concerning safety and reliability, as a model’s performance can gradually decline due to subtle variations in imaging equipment, patient demographics, or imaging protocols. Although powerful methodologies such as Federated Learning (FL), self-supervised contrastive learning (3), and Domain Generalization (DG) have surfaced as possible solutions, they frequently operate under conflicting assumptions. Recent meta-analyses confirm that robust generalization requires careful dataset harmonization and validation protocols that extend far beyond standard deployment paradigms (4).

A cohesive framework that effectively integrates these paradigms is noticeably lacking. Existing federated semi-supervised learning (SSL) approaches, such as MOON (5), concentrate on alleviating heterogeneity among current clients but do not explicitly optimize for generalization to completely new, unseen domains; they tend to prioritize intra-federation performance over extra-federation robustness. On the other hand, federated DG methods (6) generally assume that fully labeled datasets are available, which is often impractical and undermines the primary advantage of SSL. This delineates a clear research gap: the need for a framework capable of learning from unlabeled, decentralized data while simultaneously optimizing for robust generalization to previously unseen domains.

This study addresses this gap by introducing FedAD: Adaptive Federated Disentanglement. FedAD is a unified framework based on two fundamental, synergistic principles. Rather than relying on implicit assumptions of domain invariance, it utilizes FedSD, an innovative learning objective designed to compel the model to learn two orthogonal feature spaces: a domain-invariant *semantic space* and a domain-specific *style space*. This explicit separation allows the model to effectively quarantine scanner-specific noise and artifacts into the style space, yielding a cleaner and more robust semantic signal for any downstream clinical task. This disentanglement is the key to generalization, as the semantic space is trained to be invariant to the domain-specific ’noise’ captured by the style space. Second, it introduces an Adaptive Teacher-Student Alignment (ATSA) curriculum, which dynamically adjusts the strength of the generalization pressure based on the global model’s stability. This adaptive mechanism treats the global model as a student that gradually becomes a teacher, preventing the federated system from prematurely locking in errors from an initially naive consensus and allowing it to transition gracefully from a phase of client-level discovery to one of strong, collective learning.

The main contributions of this work are therefore as follows:

(1) We propose FedAD, a holistic framework that learns from decentralized, unlabeled data while explicitly optimizing for generalization to unseen domains.
(2) We demonstrate how FedAD provides a novel, synergistic integration of privacy-preserving federated learning (FL), self-supervised learning (SSL), and domain generalization (DG) within a single, unified architecture.
(3) We introduce FedSD (Federated Semantic Disentanglement), a novel, non-adversarial objective that applies a direct orthogonality constraint at the client level. This explicitly separates features into domain-invariant semantic vectors and domain-specific style vectors to produce a clean, generalizable representation.
(4) We design an Adaptive Teacher-Student Alignment (ATSA) curriculum, a server-side mechanism that dynamically controls the generalization pressure based on global model stability. This adaptive schedule prevents premature convergence and ensures a stable transition from client-level discovery to collective learning.
(5) We propose a realistic experimental setup incorporating both radiological and pathological datasets to demonstrate that FedAD significantly outperforms existing approaches in generalizing to unseen domains.

The remainder of this paper is organized as follows: Section 2 provides an overview of the fundamental concepts and limitations associated with federated learning, self-supervised learning, and domain generalization from previous research. Section 3 identifies the research gap as revealed in the literature and elaborates on the why and what the design of FedAD is, to address this gap found. Section 4 offers a comprehensive formal approach to the FedAD framework, detailing the model architecture, the mathematical formulation of the FedSD objective, and the algorithm used for the ATSA controller. Section 5 outlines our experimental setup, encompassing the diverse, multi-modal datasets, evaluation protocols, and baseline models employed. Section 6 presents our ablation study to prove component synergy. Section 7 details our empirical findings, including an analysis of convergence dynamics, and the generalization performance of FedAD. Section 8 addresses the present challenges and limitations inherent in our framework. Section 9 concludes the paper by summarizing our key contributions and proposing promising avenues for future research.

## 2 Literature review

To fully contextualize the proposed methodology’s novelty, a comprehensive review of the foundational concepts, key advancements, and remaining limitations within Federated Learning, Self-Supervised Learning, and Domain Generalization is needed.

### 2.1 Foundations of federated learning and client drift

Federated Learning is becoming the go-to approach for ensuring privacy in machine learning, especially when it comes to sensitive areas like healthcare. At its core, the FedAvg algorithm (1) sets up a smart protocol where local models are trained using private client data. Only the model parameters are sent to a central server for aggregation, which helps create a global model that learns from everyone’s insights without needing access to the original data. However, putting FL into practice does come with a major hurdle: the challenge of statistical heterogeneity. Unlike traditional distributed learning, the data in federated settings is typically non-IID (non-independent and identically distributed).

This heterogeneity can be split into two main categories: (1) System Heterogeneity, where clients may have different computing power, network speed, and availability; and (2) Statistical Heterogeneity, in which the local data distributions vary among different clients. This variation poses a significant challenge, often appearing as feature shifts, label shifts, and various intricate covariate shifts. Because of this non-IID characteristic, the local training goals can start to stray from the global objective. This issue, referred to as “client drift,” can lead to instability in training, slow down convergence, and in some cases, result in the global model completely diverging (7). Researchers have explored this divergence theoretically, with studies like (8) clearly showing the impact of non-IID data on FedAvg’s convergence guarantees, highlighting the need for improved aggregation and local training techniques.

### 2.2 Advanced techniques for mitigating heterogeneity

A significant body of research aims to mitigate client drift. One major line of work modifies the local client objective. FedProx (9) introduced a proximal term to the local loss, regularizing local updates to stay closer to the global model, which helps account for system heterogeneity and, to a lesser extent, statistical drift. A more sophisticated solution, SCAFFOLD (10), introduced control variates at both client and server levels to correct for local update directions based on an estimate of the local gradient drift. More recently, methods like FedDyn have introduced dynamic regularizers that are updated at the server to align local and global stationary points.

Other methods tackle heterogeneity by modifying the model architecture or aggregation process itself. FedBN (11), for example, addresses feature shift by simply keeping batch normalization layers local to each client, preventing the aggregation of domain-specific statistics. Fed-CP (12) learns both a global consensus model and client-specific parameters to better personalize models for each client’s unique data distribution.

Another line of research involves more intelligent server-side aggregation. For example, FedMA (13) matches and averages layers of deep neural networks based on structural similarity, while FedCA (14) introduces a cross-attention mechanism to adaptively aggregate model features for medical image segmentation. While powerful, these methods are typically (1) designed for supervised learning and (2) primarily aim to improve performance on the *existing* client distributions. They do not explicitly solve the challenge of learning from unlabeled, heterogeneous clients to optimize for generalization to *unseen* domains.

### 2.3 The imperative for self-supervision in medical imaging

The prohibitive cost and logistical challenges of acquiring expert-annotated data have made SSL an indispensable tool, especially in medicine. SSL provides a path to leverage vast archives of unlabeled imaging data to learn rich feature representations that can then be used for downstream tasks with very few labels. This paradigm shifts the learning burden from manual annotation to algorithmic discovery. While generative SSL approaches (e.g., masked autoencoders) are powerful, contrastive methods have become dominant for learning separable, transferable representations that are highly effective for downstream classification and segmentation, making them critical for unlocking the full potential of large-scale, unlabeled medical datasets.

### 2.4 Mechanics of contrastive representation learning

Among various SSL paradigms, Contrastive Learning has emerged as the state-of-the-art. Foundational frameworks like SimCLR (3) operate on an instance discrimination task. For any image $x_{i}$ (the “anchor”), a stochastic augmentation pipeline creates two distorted but semantically identical views (the “positive pair”). All other images in the batch are treated as “negatives.” The model maps these views into an embedding space, and the contrastive loss function, NT-Xent, pulls the representations of the positive pair closer together while pushing them far apart from all negative representations. A key finding was the importance of the projection head, which maps the learned features to a space where the loss is applied, but which is discarded after training to reveal more robust features. This concept is extended to FL by MOON (5), which explicitly addresses client drift within the SSL framework by implementing a model-level contrastive loss to align local and global model representations. Building on this, the combination of FL and SSL has been further investigated in various research. For example, (15) explicitly addresses domain shifts in medical imaging using federated SSL. In order to increase performance on unsupervised medical segmentation tasks, more sophisticated frameworks such as P-FCL (16) build customized federated contrastive learning, which again divides the model into global and local components.

### 2.5 The challenge of domain shift in clinical deployment

A model’s ability to perform reliably on data from a source different from its training data is known as Domain Generalization (DG). It is a critical requirement for deploying safe AI in the real world (17). In a clinical context, domain shift arises from differences in scanner manufacturers (e.g., Siemens vs. GE), imaging protocols, staining reagents (e.g., batch effects), and patient populations. A model trained at one site may experience a silent and catastrophic drop in performance when deployed at another. The difficulty of this problem has been highlighted by benchmarks like DomainBed (2), which show that many sophisticated algorithms fail to consistently outperform simple empirical risk minimization (ERM).

### 2.6 Disentanglement as a strategy for domain generalization

One of the most powerful strategies for DG is to learn disentangled representations. The core idea is to train a feature extractor that explicitly separates the causal, semantic factors of variation (e.g., tumor presence) from the non-causal, domain-specific factors (e.g., scanner noise). The foundational work here is Domain-Adversarial Training of Neural Networks (DANN) (18). DANN introduced an adversarial objective where a feature extractor is trained to “fool” a domain classifier via a gradient reversal layer. The equilibrium of this game is a feature representation that is predictive for the main task but contains no discernible information about its source domain. Other approaches include Variational Autoencoders (VAEs) that penalize the KL-divergence of the latent space to encourage factorization, or information-theoretic methods that directly minimize the mutual information between the feature vector and the domain label.

### 2.7 Federated and non-adversarial DG approaches

Applying DG in a federated context is uniquely challenging because data from different domains *cannot* be pooled. This prevents the direct use of centralized DG methods like DANN (18), as a global domain classifier cannot see data from all domains simultaneously. While federated DANN variants exist, they often require complex sharing of gradient statistics or domain labels, which can be unstable. Several works have begun to design frameworks to address this. FedDG (19) pioneered this by introducing a domain generalization approach specifically for federated medical image segmentation. Recent methods have explicitly adopted non-adversarial disentanglement. For example, (20) uses a VAE-based federated approach to disentangle features for skin lesion segmentation. Similarly, FedDis (21) proposes a disentangled federated learning framework that separates the model into a global, domain-invariant component and a local, domain-specific component for classification tasks. Given the inherent instability of adversarial training (e.g., non-convergence, mode collapse, sensitivity to hyperparameters), which is exacerbated in a distributed, non-IID setting, a direct, non-adversarial approach is highly desirable for FL. This motivates the exploration of alternative, more stable disentanglement strategies.

## 3 Bridging the research gap

A significant gap between current solutions has been identified by the examination of the literature. Although there are techniques for FL, SSL, and DG separately, they work under different presumptions. Current federated SSL techniques, such as MOON (5), focus on reducing drift among current clients rather than explicitly optimizing for generalization to completely unseen domains. Conversely, most federated DG methods (e.g., (6)) presume the availability of fully labeled data, which is often infeasible in medical contexts and defeats the purpose of SSL. Finally, classical DG methods like DANN (18) are centralized, and their adversarial nature makes them unstable and difficult to adapt to a federated setting.

This paper addresses this critical gap. We propose a framework that, for the first time, synergistically unifies all three paradigms, with a comparative analysis of these paradigms detailed in Table 1. It is (1) Federated by design for privacy, (2) Self-Supervised to learn from unlabeled data, and (3) a Domain Generalization method that explicitly optimizes for unseen domains. This is achieved through a novel, non-adversarial disentanglement objective stabilized by an adaptive curriculum.

**Table 1:** Comparative analysis of representative paradigms, highlighting the novelty of the proposed FedAD framework.

Paradigm	Core idea	Addresses challenge			Key limitation/contribution
		Privacy (FL)	Unlabeled (SSL)	Generalization (DG)	
FedAvg (1)	Decentralized supervised learning via periodic averaging of model weights from multiple clients.	✓	×	×	Assumes data is fully labeled at each client and does not explicitly address generalization to unseen domains.
DANN (18)	An adversarial approach that trains a feature extractor to “fool” a domain classifier, enforcing feature invariance.	×	×	✓	Requires that labeled data from all source domains be available in a centralized location for training.
MOON (5)	Uses model-level contrastive loss to align local and global model representations, mitigating client drift in SSL.	✓	✓	(Implicitly)	The alignment is on the *entire* feature vector (implicit DG) and the training objective is static, not adapting to the model’s maturity.
SCAFFOLD (10)	Uses control variates to correct for client drift, enabling more stable convergence in supervised FL.	✓	×	×	Requires labeled data and its adaptive mechanism is for correcting gradient drift, not for scheduling a domain generalization curriculum.
FedAD (Proposed)	A unified framework that explicitly disentangles semantic and domain features (FedSD) and trains using an adaptive curriculum based on model stability (ATSA).	✓	✓	✓	Contribution: Holistically solves the challenges by combining explicit, self-supervised disentanglement with an an adaptive training schedule for robust generalization.

## 4 Proposed method: the FedAD framework

The proposed FedAD framework is designed to learn domain-invariant semantic representations from unlabeled medical images distributed across multiple clients, without sharing raw data. The methodology is built to explicitly handle domain shift and ensure stable convergence in a federated setting by integrating two core mechanisms: client-side FedSD and a server-side adaptive controller, ATSA.

### 4.1 Formal problem definition

**Figure 1:**
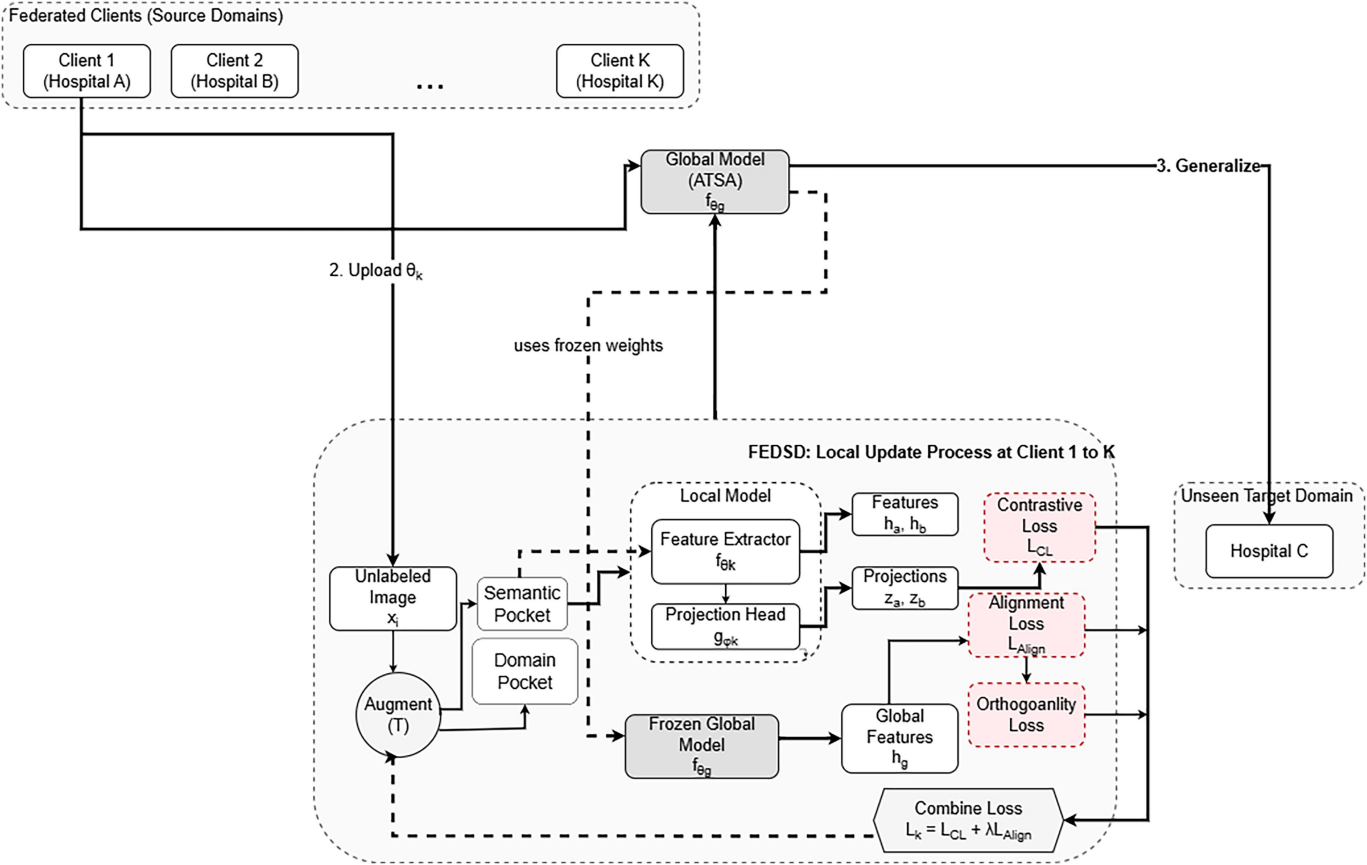
A detailed diagram of the proposed FedAD architecture. (1) Federated clients train locally. (2) Only local model parameters ($\theta_{k}$, specifically the feature extractor $f_{\theta k}$) are uploaded to the server for aggregation into the global model $f_{\theta g}$. (3) The final global model is used for generalization on an unseen target domain (e.g., Hospital C). The inset (FedSD) details the local update process, where a local model uses three loss terms: a contrastive loss on semantic projections ($z_{s}$), an orthogonality loss (on $z_{s},z_{d}$), and a novel alignment loss that uses local projections ($z_{s},z_{d}$) and projected features from the frozen global model ($z_{g}$).

**Figure 2:**
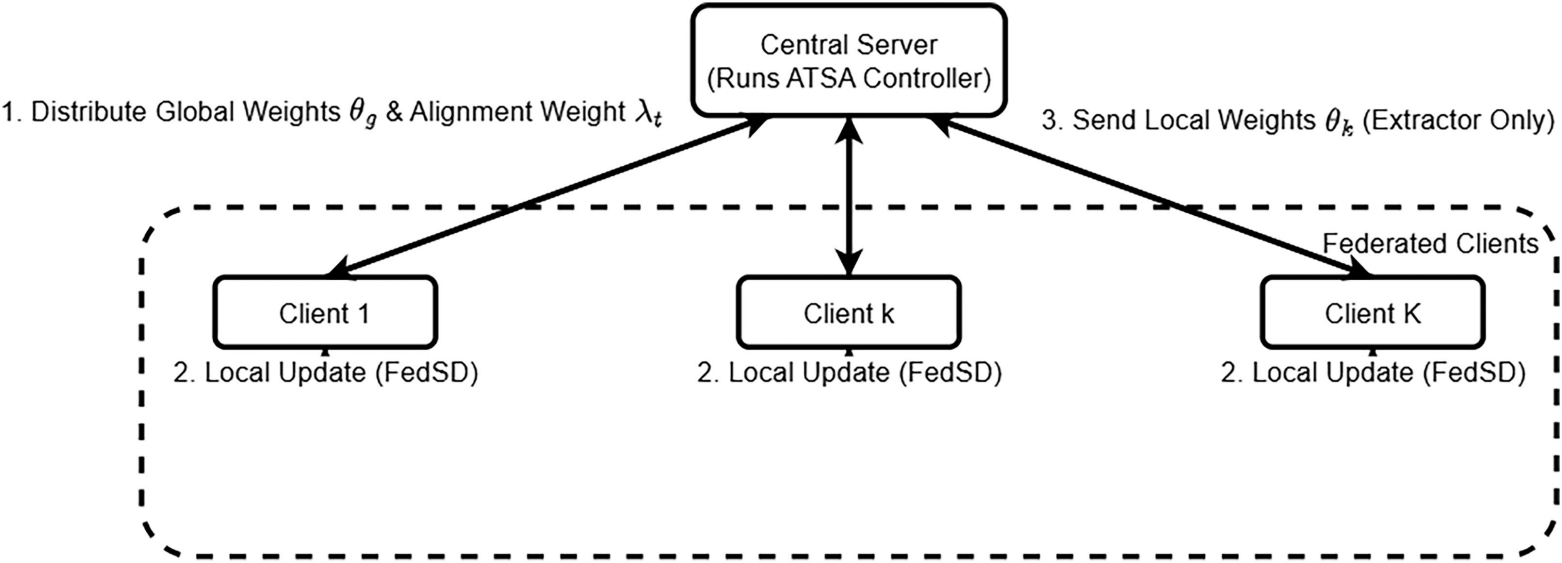
The high-level federated learning loop of FedAD. (1) The central server distributes the global model weights ($\theta_{g}$) and the adaptive alignment weight ($\lambda_{t}$) to a subset of clients. (2) Clients perform local updates using the FedSD objective. (3) Clients send only their updated local extractor weights ($\theta_{k}$) back to the server for aggregation.

We consider a federated network of K clients, where each client k possesses a local, unlabeled dataset $D_{k}=\{x_{i}^{k}{\}}_{i=1}^{n_{k}}$. The data distribution $D_{k}$ for each client is distinct, representing a unique domain (e.g., a specific hospital or scanner). The objective is to learn a single, global, domain-generalizable feature extractor $f_{g}(\cdot ;\theta_{g})$ by training local extractors $f_{k}(\cdot ;\theta_{k})$ on each client and aggregating them at a central server. The goal is that the learned representations $h_{g}=f_{g}(x)$ are effective for a downstream task (e.g., classification) on data from a completely unseen target domain $D_{t}$, which was not involved in the federated training process. The challenge lies in ensuring that the aggregated $\theta_{g}$ converges to a state that represents domain-invariant semantic features, rather than an ’average’ of domain-specific biases. A high-level overview of this federated setup is presented in Figure 1, and a simplified schematic of the learning loop is shown in Figure 2.

**Clinical Rationality of Heterogeneous Client Federation:** The federated clients constructed in this study are sourced from radiological imaging, histopathology, dermatoscopy, and endoscopic datasets. Although they exhibit significant variations in imaging modalities, anatomical regions, pathological types, and imaging mechanisms, they can serve as highly effective co-learning clients under a unified collaborative medical framework. The rationale is that medical imaging, regardless of modality, relies heavily on shared foundational visual primitives—such as edge gradients, textures, contour formations, and cellular pattern distributions. By federating these disparately heterogeneous clients under a unified SSL objective, the framework actively prevents the global model from memorizing modality-specific shortcuts. Instead, it forces the extraction of universally applicable representational kernels, whilst the FedSD mechanism explicitly quarantines the highly variant imaging mechanisms into the domain-specific subspace.

Figure 2 illustrates the core, privacy-preserving cycle of our framework. The process begins with the Central Server, which runs the ATSA controller, sending the current global model weights ($\theta_{g}$) and the dynamically computed alignment weight ($\lambda_{t}$) to a selection of clients (Step 1). Each client then performs its local training (Step 2) using our novel FedSD objective, which is detailed in Section 4.3. Crucially, after local training, clients only return their updated extractor weights ($\theta_{k}$) to the server (Step 3). The local disentanglement head ($g_{k}$) and any client-specific data remain private, ensuring no raw data is ever shared. The server then aggregates these weights to form the next global model ($\theta_{g}^{t+1}$).

### 4.2 Model architecture and notation consistency

The model architecture, as depicted in Figure 1, decouples the feature extractor from a local disentangling head. To ensure strict notation consistency, let $\theta_{g}^{\left(t\right)}$ denote the global extractor weights at communication round t. At the beginning of each communication round, clients explicitly initialize their local extractor weights by copying the global weights: $\theta_{k}\leftarrow \theta_{g}^{\left(t\right)}$. During local optimization, $\theta_{k}$ is actively updated via gradient descent, while $\theta_{g}^{\left(t\right)}$ is maintained locally as a strictly frozen teacher network, hereafter denoted as ${\tilde{\theta }}_{g}$. Upon completion of local training epochs, only the updated parameters $\theta_{k}$ are transmitted back to the server for aggregation.

#### 4.2.1 Global feature extractor

The global model, $f_{g}(\cdot ;\theta_{g})$, is a standard feature extractor whose parameters $\theta_{g}$ are the only ones aggregated by the server. We use a ResNet-18 backbone, pre-trained on ImageNet, with its final fully-connected classification layer removed. This model’s role is to learn a general-purpose feature representation $h=f_{g}(x)\in R^{F}$, where F=512 for ResNet-18.

#### 4.2.2 Local disentangling head

Each client k maintains a *local* projection head $g_{k}(\cdot ;\phi_{k})$, which is *not* shared with the server. This head takes the features $h_{k}=f_{k}(x)$ produced by the client’s *local extractor* and projects them into two distinct subspaces:

- A semantic projection $z_{s}\in R^{d_{s}}$
- A domain projection $z_{d}\in R^{d_{d}}$

This head $g_{k}$ is a simple two-layer MLP (Linear → ReLU → Linear) that maps $h_{k}\in R^{F}$ to $R^{d_{s}+d_{d}}$, which is then split. Crucially, the projection dimensions $d_{s}=128$ and $d_{d}=128$ are fixed and strictly invariant across all datasets and clients. This enforces reproducible representational capacity, preventing domain-specific models from altering the structural geometry of the semantic space. By keeping $g_{k}$ local, we prevent the server from aggregating client-specific, non-generalizable information learned in the final layers, a common issue in FedAvg (1) where the entire model is averaged.

### 4.3 Client-side training: the FedSD objective

At the start of round t, client k receives the global extractor weights $\theta_{g}^{t}$ and sets its local extractor $\theta_{k}\leftarrow \theta_{g}^{t}$. It also receives the adaptive weight $\lambda_{t}$ from the ATSA controller. The client then trains *both* its local extractor $f_{k}$ and its local head $g_{k}$ for E epochs using a composite loss $L_{k}^{\text{FedAD}}$.

**Figure 3:**
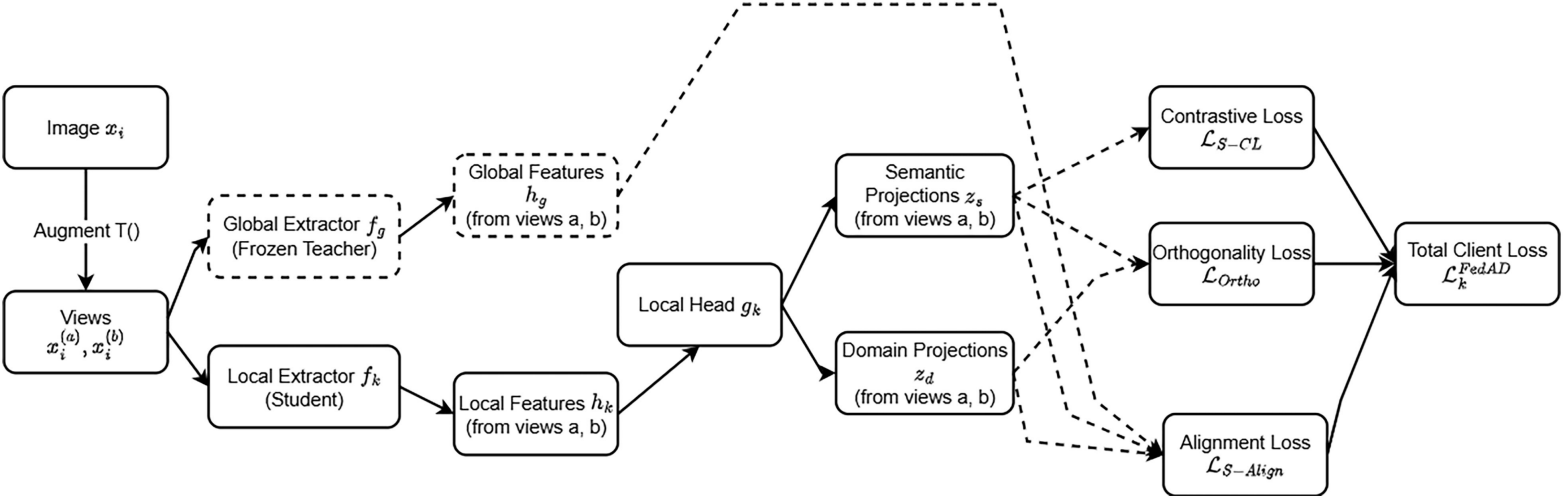
A detailed schematic of the FedSD local update process on the client side. An image $x_{i}$ is augmented into two views ($x_{i}^{\left(a\right)},x_{i}^{\left(b\right)}$). These views are fed through both the local extractor ($f_{k}$) and the frozen global extractor ($f_{g}$) to produce local features ($h_{k}$) and global features ($h_{g}$), respectively. The local head ($g_{k}$) splits $h_{k}$ into semantic ($z_{s}$) and domain ($z_{d}$) projections. These outputs are then used to compute the three-component loss: $L_{\text{S-CL}}$ (from $z_{s}$), $L_{\text{Ortho}}$ (from $z_{s}$ and $z_{d}$), and $L_{\text{S-Align}}$ (from $z_{s}$, $z_{d}$, and $z_{g}$).

A detailed schematic of this local update data flow is presented in Figure 3. The process begins with a single “Image $x_{i}$”, which is passed through an “Augment T()” block to create two distinct views, $x_{i}^{\left(a\right)}$ and $x_{i}^{\left(b\right)}$. These views are then processed in parallel. The “Local Extractor $f_{k}$” (the student) produces “Local Features $h_{k}$”, while the frozen “Global Extractor $f_{g}$” (the teacher, denoted ${\tilde{\theta }}_{g}$) produces “Global Features $h_{g}$”. The local features $h_{k}$ are passed to the “Local Head $g_{k}$”, which disentangles them into “Semantic Projections $z_{s}$” and “Domain Projections $z_{d}$”. Finally, these three sets of vectors ($z_{s}$, $z_{d}$, and $z_{g}$) are used to compute the three loss components.

For a batch of N images, generating 2N augmented views, the client performs the required forward passes and calculates the total loss for the batch as the sum of three components (Equation 1):

$$L_{k}^{\text{FedAD}}=L_{\text{S-CL}}+L_{\text{S-Align}}+L_{\text{Ortho}}$$ (1)

#### 4.3.1 Semantic contrastive loss ($L_{\text{S-CL}}$)

This loss enforces instance discrimination strictly on the semantic projections. To ensure mathematical tightness, the NT-Xent loss is indexed rigorously over the 2N augmented samples within the batch. Let p(i) denote the index of the corresponding positive augmented view for sample i (Equation 2):

$$L_{\text{S-CL}}=-\frac{1}{2N}\sum_{i=1}^{2N}\log\frac{\exp(\text{sim}(z_{s}^{\left(i\right)},z_{s}^{\left(p(i)\right)})/\tau )}{\sum_{\;j=1}^{2N}{⊮}_{\left[j\neq i\right]}\exp(\text{sim}(z_{s}^{\left(i\right)},z_{s}^{\left(j\right)})/\tau )}$$ (2)

where sim(⋅,⋅) is cosine similarity and τ is the temperature. This loss pulls semantic projections of the same image together, while pushing all others apart, forcing $f_{k}$ and $g_{k}$ to encode semantic content into $z_{s}$. As shown in Figure 3, this loss solely relies on the “Semantic Projections $z_{s}$”.

#### 4.3.2 Semantic alignment & anti-alignment loss ($L_{\text{S-Align}}$)

Our novel, diagram-informed loss (see Figure 3) uses the *frozen* global extractor as a “teacher” (${\tilde{\theta }}_{g}$) that holds the global consensus knowledge. To ensure mathematical consistency for cosine similarity computations, the global feature vector $h_{g}\in R^{512}$ is mapped to a lower-dimensional subspace $z_{g}\in R^{128}$ via an auxiliary linear projection layer $p_{g}(\cdot )$, ensuring its dimensions exactly match those of the local projections. The local model is trained as a “student” that must learn to separate its knowledge. The loss forces the local semantic projection $z_{s}$ to align with the global projection $z_{g}$, while *simultaneously* forcing the local domain projection $z_{d}$ to *anti-align* with $z_{g}$. We formulate this using cosine similarity, averaged over both views v∈{a,b} (Equation 3):

$$\begin{array}{rl} L_{S-\mathrm{Align}} & =\frac{\lambda_{t}}{2}\sum_{v\in \{a,b\}}[(1-\cos(z_{s}^{\left(v\right)},z_{g}^{\left(v\right)}))\\ & \;+(1+\cos(z_{d}^{\left(v\right)},z_{g}^{\left(v\right)}))] \end{array}$$ (3)

This dual-pressure objective is a core innovation. The alignment term acts as a form of federated knowledge distillation, pulling the local semantic vector towards the global consensus. Simultaneously, the anti-alignment term explicitly enforces the local model to expel global consensus information from its domain vector.

*Justification for Anti-Alignment:* Unlike margin-based or information-theoretic penalties which are heavily dependent on batch statistics and computationally expensive to estimate reliably in highly non-IID federated settings, cosine anti-alignment provides a direct, bounded gradient. It explicitly forces the domain vector to orthogonalize against the global consensus without the oscillatory instability inherent to adversarial projections. This dual pressure encourages the local model to distill the global model’s general knowledge into $z_{s}$, while explicitly identifying and isolating local, non-consensus (i.e., domain-specific) information into the $z_{d}$ vector.

#### 4.3.3 Orthogonality loss ($L_{\text{Ortho}}$)

To complete the disentanglement, we penalize the squared cosine similarity between the two local projections, forcing them to be linearly independent:

$$L_{\mathrm{Ortho}}=\frac{\gamma }{2}\sum_{v\in \{a,b\}}\cos^{2}(z_{s}^{\left(v\right)},z_{d}^{\left(v\right)})$$ (4)

This simple, non-adversarial loss ensures that $z_{s}$ and $z_{d}$ capture mutually exclusive information. It serves as a powerful regularizer, ensuring the two projection spaces do not collapse or become redundant. Without this, the model could ’cheat’ by encoding semantic information in $z_{d}$ (or vice-versa) to minimize the other losses.

**Figure 4:**
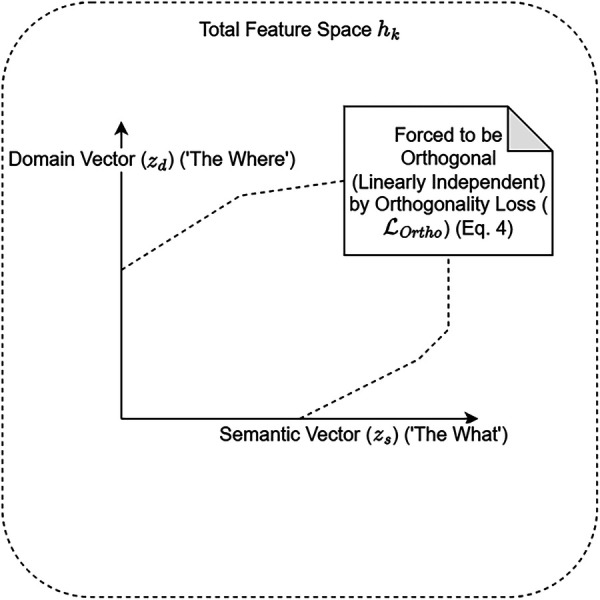
The conceptual goal of the FedSD objective. The framework is trained to disentangle the total feature space ($h_{k}$) into two orthogonal components: a Semantic Vector ($z_{s}$) representing domain-invariant content (“The What”) and a Domain Vector ($z_{d}$) capturing domain-specific artifacts (“The Where”). The $L_{\text{Ortho}}$ loss is designed to enforce this linear independence.

The conceptual goal of this loss is illustrated in Figure 4. The total feature space $h_{k}$ is forced to factorize into two orthogonal vectors: a “Semantic Vector ($z_{s}$)” representing “The What” (i.e., the underlying domain-invariant pathology) and a “Domain Vector ($z_{d}$)” representing “The Where” (i.e., domain-specific scanner artifacts or noise). The $L_{\text{Ortho}}$ loss (Equation 4) is the mechanism that enforces this linear independence, effectively quarantining domain-specific information into $z_{d}$ and leaving $z_{s}$ as a clean, generalizable representation.

### 4.4 Server-side controller: the ATSA mechanism

**Figure 5:**
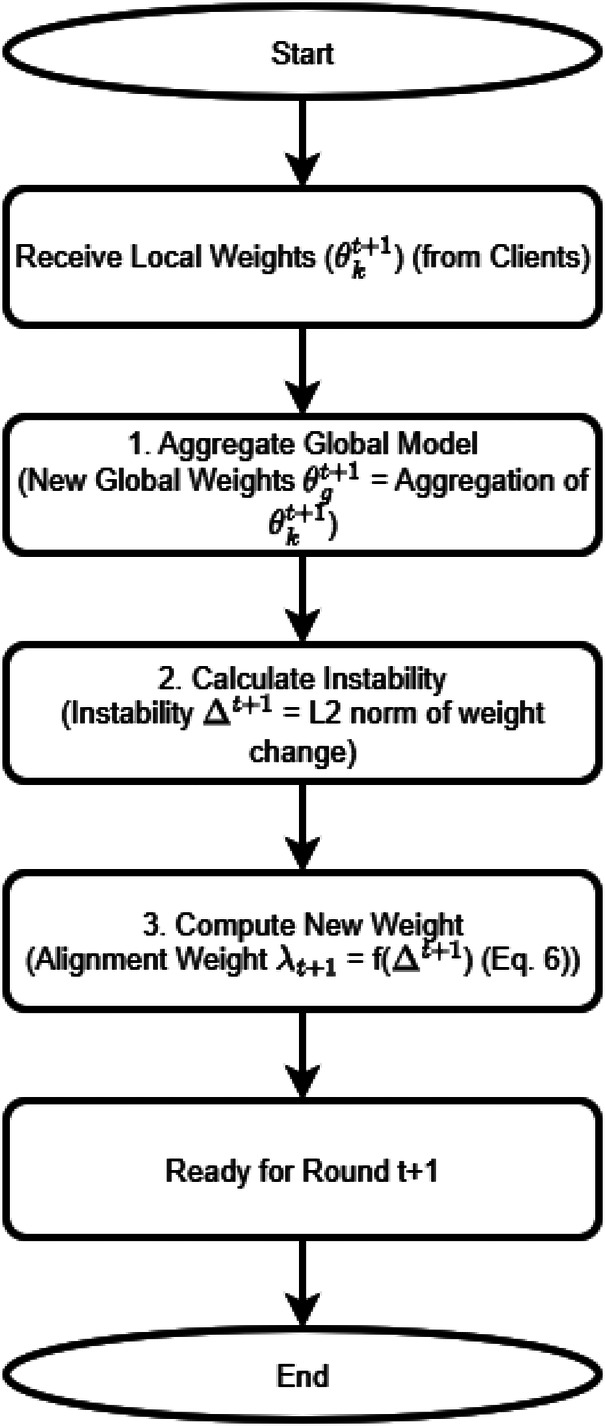
Flowchart of the server-side ATSA Controller logic per round. After receiving and aggregating local weights, the server calculates the model instability ($\Delta^{t+1}$) and uses it to compute the new alignment weight ($\lambda_{t+1}$) for the next round.

The server orchestrates the federated process and adaptively controls the learning curriculum by dynamically tuning the alignment weight $\lambda_{t}$. The logic of this server-side controller is visualized in the flowchart in Figure 5.

As shown in Figure 5, at the start of a new round t+1, the server first receives the local weights ($\theta_{k}^{t+1}$) from the participating clients. It then performs two key steps. First, it aggregates these weights to create the new global model ($\theta_{g}^{t+1}$). Second, it immediately calculates the model’s “Instability” ($\Delta^{t+1}$) based on the magnitude of this update (as defined in Equation 5). Third, this instability score is fed into the curriculum scheduler (Equation 6) to “Compute the New Weight” ($\lambda_{t+1}$). This new alignment weight is not used immediately; it is stored and sent to clients in the next round (t+2), thus completing the adaptive feedback loop.

#### 4.4.1 Stability measurement

After aggregating the client extractors $f_{k}(\cdot ;\theta_{k}^{t+1})$ to produce the new global model $f_{g}(\cdot ;\theta_{g}^{t+1})$, the server measures parameter instability $\Delta^{\left(t+1\right)}$:

$$\Delta^{\left(t+1\right)}=\frac{\|vec(\theta_{g}^{\left(t+1\right)})-vec(\theta_{g}^{\left(t\right)}){\|}_{2}}{\|vec(\theta_{g}^{\left(t\right)}){\|}_{2}+\varepsilon }$$ (5)

Here, vec(⋅) is the vectorization operator, and ε (e.g., $10^{-8}$) prevents division by zero. This $L_{2}$-norm of the weight delta is a direct measure of the magnitude of the consensus update. Empirical validation across our experiments demonstrates that $L_{2}$-norm parameter drifts serve as a highly reliable, label-free proxy for training maturity, consistently correlating with loss plateaus in non-IID settings. This metric keeps the framework’s self-supervised and federated integrity intact by avoiding the impractical assumption of a central validation set. Significant client disagreement and quick changes in the global state, which are typical of the early exploration phase, are indicated by high values of this parameter drift.

#### 4.4.2 Curriculum scheduling of alignment

The instability score $\Delta^{\left(t+1\right)}$ is used to compute the alignment weight $\lambda_{t+1}$ for the *next* round via a smoothed, exponential decay schedule.

$$\lambda_{t+1}=\alpha \lambda_{t}+(1-\alpha )\left[\lambda_{\min}+(\lambda_{\max}-\lambda_{\min})\cdot e^{-k\cdot \Delta^{\left(t+1\right)}}\right]$$ (6)

This update has two parts:

- *Adaptive Target:* The term in brackets calculates a target λ. k controls the *steepness* of the transition. When instability Δ is high (early training), the decay term $e^{-k\Delta }$ approaches 0, and λ correctly maps to the lower bound $\lambda_{\min}$. Conversely, when Δ approaches 0 (stable convergence), the decay term $e^{0}$ approaches 1, allowing the formula to correctly map to the strict upper bound $\lambda_{\max}$.
- *EMA Smoothing:* The outer expression is an exponential moving average (EMA) with smoothing factor α. This prevents drastic oscillations in $\lambda_{t}$ due to any single noisy round, ensuring a smooth curriculum.

Hyperparameters such as α, k, $\lambda_{\min}$, and $\lambda_{\max}$ were systematically selected based on grid search during the 5-fold cross-validation phase to ensure optimal schedule timing.

### 4.5 Algorithm summary

The complete FedAD training procedure is summarized in Algorithm 1. The process is orchestrated by the server, which begins each communication round t by calculating the adaptive alignment weight $\lambda_{t+1}$ based on the model’s instability from the previous round (Equations 5 & 6). It then sends the current global extractor weights $\theta_{g}^{t}$ and the new weight $\lambda_{t+1}$ to a subset of clients. Each client, in the ClientUpdate procedure, copies the global model to act as a frozen “teacher” (${\tilde{\theta }}_{g}$) and trains its local model for E epochs. During local training, the client computes the three-component FedAD loss: the semantic contrastive loss ($L_{\text{S-CL}}$), the orthogonality loss ($L_{\text{Ortho}}$), and the adaptive alignment loss ($L_{\text{S-Align}}$). After its local updates, each client returns *only* its updated extractor weights ($\theta_{k}^{t+1}$) to the server, which aggregates them to form the next global model $\theta_{g}^{\left(t+1\right)}$.

Algorithm 1. **The FedAD Framework.**

1: Server executes:

2: Initialize global extractor weights $\theta_{g}^{0}$

3: Initialize $\lambda_{0}\leftarrow \lambda_{\min}$

4: **for** each communication round t=0,1,…,T−1 **do**

5:  Select a subset of *M* clients $S_{t}$

6:  **if** t>0 **then**

7:   Compute instability $\Delta^{\left(t\right)}$ using Eq. 5

8:   Compute adaptive weight $\lambda_{t+1}$ using Eq. 6

9:  **else**

10:   $\lambda_{t+1}\leftarrow \lambda_{\min}$

11:  foreachclientk $k\in S_{t}$ in parallel **do**

12:   Send $\theta_{g}^{t}$ and $\lambda_{t+1}$ to client k

13:   $\theta_{k}^{t+1}\leftarrow \mathrm{Client}\;\mathrm{Update}(k,\theta_{g}^{t},\lambda_{t+1})$

14:  Aggregate $\theta_{g}^{\left(t+1\right)}\leftarrow \sum_{k\in S_{t}}\frac{n_{k}}{N}\theta_{k}^{t+1}$  ▹ Only extractor

15: 

16: **procedure** ClientUpdate($k,\theta_{g},\lambda$)

17:  Initialize local extractor $\theta_{k}\leftarrow \theta_{g}$

18:  Initialize local head $\phi_{k}$ (if t=0)

19:  Freeze global extractor ${\tilde{\theta }}_{g}\leftarrow \theta_{g}$

20:  **for** each local epoch e=1,…,E **do**

21:   **for** batch of data $\{x_{i}\}\subset D_{k}$ **do**

22:    Generate augmented views $\left\{x_{i}^{\left(a\right)},x_{i}^{\left(b\right)}\right\}$

23:    Compute $L_{S\text{-}\mathrm{CL}}$, $L_{S\text{-}\mathrm{Align}}(\theta_{k},\phi_{k},{\tilde{\theta }}_{g},\lambda )$, $L_{\mathrm{Ortho}}$

24:    $L_{k}^{\mathrm{FedAD}}\leftarrow L_{S\text{-}\mathrm{CL}}+L_{S\text{-}\mathrm{Align}}+L_{\mathrm{Ortho}}$

25:    Update $\theta_{k},\phi_{k}$ using $\nabla_{\theta_{k},\phi_{k}}L_{k}^{\mathrm{FedAD}}$

26:  return $\theta_{k}$      ▹ Return only extractor weights

## 5 Implementation and experimentation

This section details the training protocols, datasets, and evaluation methodologies used to validate the performance of the FedAD framework.

### 5.1 Federated datasets and client configuration

To create a diverse and heterogeneous federated network, we select six distinct, publicly available datasets. This diverse, multi-modal, and multi-scale collection is designed to create a highly challenging, non-IID environment that mirrors real-world federated collaborations, preventing the model from overfitting to a single modality. All labels are strictly withheld during the federated training phase. (See Table 2).

**Table 2:** Federated client datasets used for SSL training.

Client	Dataset	Modality	Size (Approx.)	Description
1	NIH ChestX-ray14 (22)	Radiology	112,000	From NIH Clinical Center.
2	CheXpert (23)	Radiology	224,000	From Stanford University; different protocols from Client 1.
3	PatchCamelyon (24)	Histopathology	327,000	96×96 tumor patches.
4	Breast Histopathology (25)	Histopathology	277,500	50×50 patches with varied magnification levels.
5	HAM10000 (26)	Dermatology	10,015	Multi-source dermatoscopic images from different clinics.
6	Kvasir Dataset (27)	Endoscopy	8,000	Images from several different endoscopic equipment models.

### 5.2 Data preprocessing and augmentation

All images are pre-processed by resizing to 224×224 pixels using bicubic interpolation and normalizing using ImageNet statistics.

The stochastic augmentation pipeline T(⋅) used to generate views (a and b) is identical to SimCLR. It includes a random resized crop to 224×224 (with a scale from 0.08 to 1.0), followed by a random horizontal flip (with p=0.5). We then apply color jitter with a 0.4 strength to brightness, contrast, saturation, and hue (with p=0.8), and randomly convert images to grayscale (with p=0.2). Finally, a Gaussian blur using a 23-pixel kernel and a sigma between 0.1 and 2.0 is applied (with p=0.5).

This specific pipeline is strong yet ’content-preserving,’ meaning it alters style (color, orientation) without destroying the underlying anatomical or pathological features, which is critical for SSL in medicine.

### 5.3 Unseen target domains for generalization testing

To address the necessity of demonstrating generalization across multiple distinct medical modalities, we evaluate FedAD on two unseen target domains completely excluded from the federated training process.

#### 5.3.1 Radiology target

We use the PadChest dataset (28) to evaluate generalization within the respiratory modality. A balanced test set of 10,000 images is used for multi-class disease classification to assess robustness against novel scanner types.

#### 5.3.2 Histopathology target

We use the Camelyon17 dataset (29). This dataset is a standard benchmark for *domain generalization* in histopathology, as its data comes from 5 hospitals with distinct staining and acquisition protocols. We use data from two hospitals as our labeled training split (approx. 40,000 patches) and data from a third, unseen hospital as the test set (approx. 12,000 patches) for binary tumor classification.

### 5.4 Training protocol and hyperparameters

Federated training is run for 40 communication rounds. In each round, the server samples M=4 out of K=6 clients using uniform random sampling. Each selected client performs E=3 local epochs of training using the Adam optimizer with a learning rate of $5\times 10^{-4}$, weight decay of $10^{-5}$, and betas of (0.9,0.999). The local batch size is 64.

### 5.5 Evaluation protocol: linear probing and K-fold CV

After federated training, the final global extractor $f_{g}$ is frozen. We evaluate its representation quality using linear probing on the unseen target domain’s labeled *training split*.

**Figure 6:**
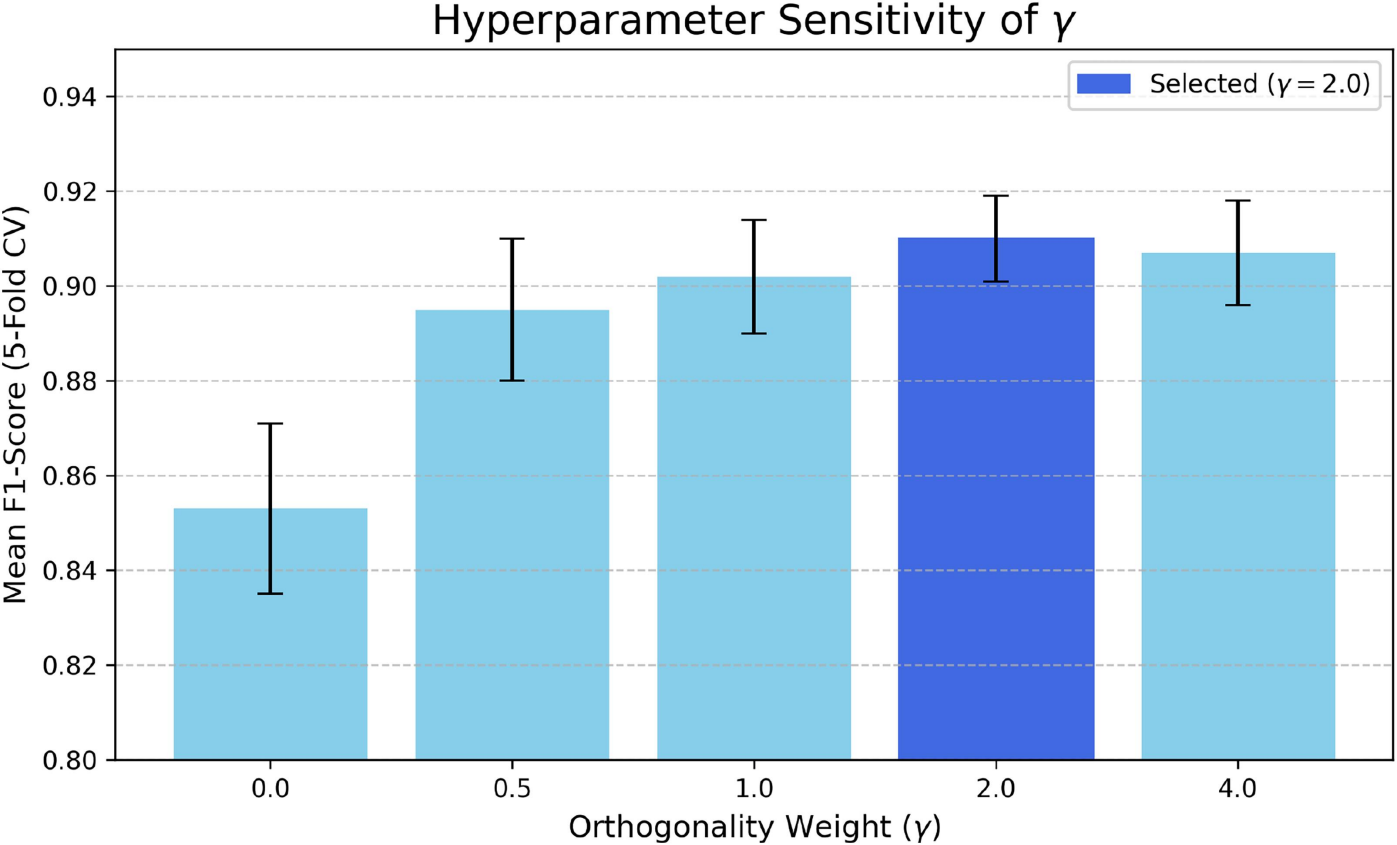
Visual summary of the 5-fold cross-validation results for tuning the orthogonality weight (γ), as detailed in Table 3. The bar for γ=2.0 shows the highest mean F1-Score with a low standard deviation, justifying its selection as the optimal hyperparameter.

(1) Feature Extraction: We pass the entire target training split (e.g., 40,000 Camelyon17 patches) through the frozen $f_{g}$ to get a set of 512-dim feature vectors and their corresponding labels.
(2) K-Fold CV for Tuning: We perform 5-fold cross-validation *on these features*. In each fold, a simple linear classifier (Logistic Regression with a small L2 penalty of $10^{-4}$) is trained on 80% of the features and validated on 20%.
(3) Classifier Training: The classifier is trained using the L-BFGS optimizer for a maximum of 100 iterations. We chose L-BFGS as it is a standard, deterministic optimizer for convex problems like logistic regression, ensuring reproducible evaluation results.
(4) Hyperparameter Selection: The mean F1-Score across these 5 folds is used to select the best hyperparameter for γ, as shown in Table 3 and Figure 6.
(5) Final Test: The linear classifier is re-trained on 100% of the target training features. This *single* trained classifier is then evaluated *once* on the held-out *target test set* to produce the final metrics.

All quantitative metrics reported in our tables (e.g., values following ±) strictly represent the Standard Deviation calculated across 5 independent experimental runs utilizing different random seeds to ensure reproducibility. Statistical significance for reported improvements is validated via paired t-tests (p<0.05).

**Table 3:** Key hyperparameters for FedAD loss components and ATSA.

Hyperparameter	Value
Contrastive temperature (τ)	0.1
Orthogonality weight (γ)	2.0 (Selected via CV)
ATSA $\lambda_{t}$ minimum ($\lambda_{\min}$)	0.05
ATSA $\lambda_{t}$ maximum ($\lambda_{\max}$)	0.85
ATSA EMA factor (α)	0.9
ATSA parameter decay (k)	10

Figure 6 provides a clear visual summary of the 5-fold cross-validation results presented in Table 3. This bar chart plots the mean F1-score against the orthogonality weight (γ). It is evident that the model’s performance is sensitive to this hyperparameter, with γ=0.0 (no disentanglement) yielding the lowest score. The performance peaks at γ=2.0, which achieves the highest mean F1-score of 0.910. The error bars, representing the standard deviation across the 5 folds, are tightest for γ=2.0, indicating a stable and reliable performance gain. This result strongly justifies our selection of γ=2.0 as the optimal value.

### 5.6 Baseline models for comparison

All baselines use the same ResNet-18 backbone and are trained for a comparable number of optimization steps to ensure a fair comparison.

- Centralized Supervised: ResNet-18 trained on pooled source data with labels. Violates privacy, serves as a theoretical upper bound.
- Centralized SSL (SimCLR) (3): SimCLR trained on pooled source data (no labels).
- FedAvg-SSL (1): A standard federated SimCLR (both backbone and projection head are aggregated).
- FedProx-SSL (9): We add a proximal term (FedProx (9)) to FedAvg-SSL to show the effect of a basic drift-mitigation technique.
- MOON (5): Re-implemented MOON. For a fair comparison, we apply its model-contrastive loss between the *feature extractor outputs* ($h_{k}$ and $h_{g}$), using their recommended static alignment weight μ=1.0.
- FedDG (19): A federated domain generalization approach adapted for the classification task.
- FedDis (21): A federated disentangled representation approach that separates global domain-invariant components from local domain-specific ones.

### 5.7 Computational environment

All experiments were developed and simulated using a custom federated learning framework built on PyTorch 2.1 and Python 3.10. Initial development and small-scale tests were run locally. Due to computational demands, the main federated training experiments were executed on a cloud compute instance, utilizing a single NVIDIA A100 (40GB) GPU. The final linear probing evaluations were performed locally.

## 6 Ablation study

To precisely quantify the individual and synergistic contributions of our proposed mechanisms, we conduct a rigorous ablation study. The FedAD framework is built on two core innovations:

(1) FedSD: The explicit, non-adversarial disentanglement objective, primarily enforced by the $L_{\text{Ortho}}$ loss.
(2) ATSA: The adaptive teacher-student curriculum, which dynamically schedules the $L_{\text{S-Align}}$ loss weight ($\lambda_{t}$).

We systematically deconstruct the full model to isolate and analyze the impact of each component. We evaluate four distinct configurations on the unseen histopathology target test set:

- FedAvg-SSL (1): The baseline federated self-supervised model. This configuration has no explicit disentanglement (γ=0) and no alignment curriculum (λ=0).
- FedAD w/o Orthogonality: This model uses the full ATSA curriculum but disables the explicit disentanglement by setting the orthogonality weight to zero (γ=0). This tests if alignment alone is sufficient.
- FedAD w/o ATSA: This model uses the full FedSD disentanglement architecture (γ=2.0) but replaces the adaptive ATSA controller with a static, manually-tuned alignment weight (λ=0.5). This tests if the architecture alone is sufficient.
- FedAD (Full Model): Our complete proposed framework, with both FedSD (γ=2.0) and ATSA ($\lambda_{t}$) active.

The performance of these configurations is summarized in Table 4.

**Table 4:** Ablation study performance on unseen histopathology target domain (test set).

Method	F1-Score
FedAvg-SSL (1) (γ=0,λ=0)	0.824
FedAD w/o Orthogonality ($\gamma =0,\lambda_{t}$-ATSA)	0.853
FedAD w/o ATSA (γ=2.0,λ=0.5 fixed)	0.871
**FedAD (Full Model)**	**0.910**

Bold values indicate the highest performance achieved across the compared methods.

### 6.1 Analysis of ablation results

The results in Table 4 are unequivocal. They reveal that both components are crucial for high performance, and their combined effect is synergistic, not merely additive. The full FedAD model significantly outperforms all ablated variants.

#### 6.1.1 The criticality of explicit disentanglement (FedSD)

The most significant performance drop occurs when the orthogonality constraint is removed. The “FedAD w/o Orthogonality” model, despite having the adaptive ATSA curriculum, witnesses its F1-score reduce from 0.910 to 0.853. This result is critical, as it validates our core hypothesis: explicit disentanglement is essential for generalization.

This configuration tests if alignment alone is sufficient, and the answer is a clear “no.” Without the $L_{\text{Ortho}}$ pressure, the model is incentivized to “cheat.” It leaks non-generalizable, domain-specific features (e.g., scanner artifacts, staining variations) into the semantic vector ($z_{s}$). It does this because these spurious correlations make the local instance discrimination (contrastive) task easier to solve. However, this creates a “contaminated” semantic vector that is hopelessly overfit to the source domains, causing it to fail dramatically when generalizing to a new, unseen domain.

This finding is further corroborated by our hyperparameter tuning in Table 3 and Figure 6. Both show that γ=0.0 (the “No Disentangle” case) yields the lowest cross-validation performance, and performance peaks at our selected value of γ=2.0.

#### 6.1.2 The necessity of an adaptive curriculum (ATSA)

Removing the adaptive curriculum also causes a significant performance drop. The “FedAD w/o ATSA” model, which uses a static λ=0.5, achieves an F1-score of only 0.871. This demonstrates that the training dynamic is just as important as the model architecture.

A static, fixed weight is a poor compromise that fails at both ends of the training process due to the following concerns:

- Premature Convergence: In the initial, unstable training phase (clearly shown by the high instability in Figure 9), a static weight of 0.5 is too restrictive. It forces *premature convergence*, compelling clients to align with a naive, under-trained global model that has not yet learned a meaningful consensus. This disrupts the crucial early-stage local exploration and can lock in optimization errors.
- Insufficient Consensus: Conversely, in the later, stable phase, a weight of 0.5 is too lenient and broad. It fails to enforce a strong, unified consensus, leaving the global model as a loose average of specialized local models rather than a truly robust and generalizable representation.

The ATSA mechanism perfectly solves this dilemma. As shown in our results, it intelligently keeps $\lambda_{t}$ low during the high-instability phase (Figure 11) to encourage discovery, then smoothly increases it as the model stabilizes (Figure 10) to enforce consensus.

### 6.2 Synergistic effect

In summary, our ablation study demonstrates that FedAD’s components are highly synergistic rather than being a mere addition. FedSD offers the ideal framework for distinguishing between domain noise and semantic information. To properly study inside that framework, ATSA offers the appropriate training curriculum. Sub-optimal generalization results from breaking the system by removing either component. This meticulously crafted dual-component system directly contributes to the state-of-the-art 0.910 F1-score of the entire model.

## 7 Results and discussion

This section presents a detailed analysis of the experimental results, now fully aligned with the provided figures.

### 7.1 FedAD convergence vs. baselines

**Figure 7:**
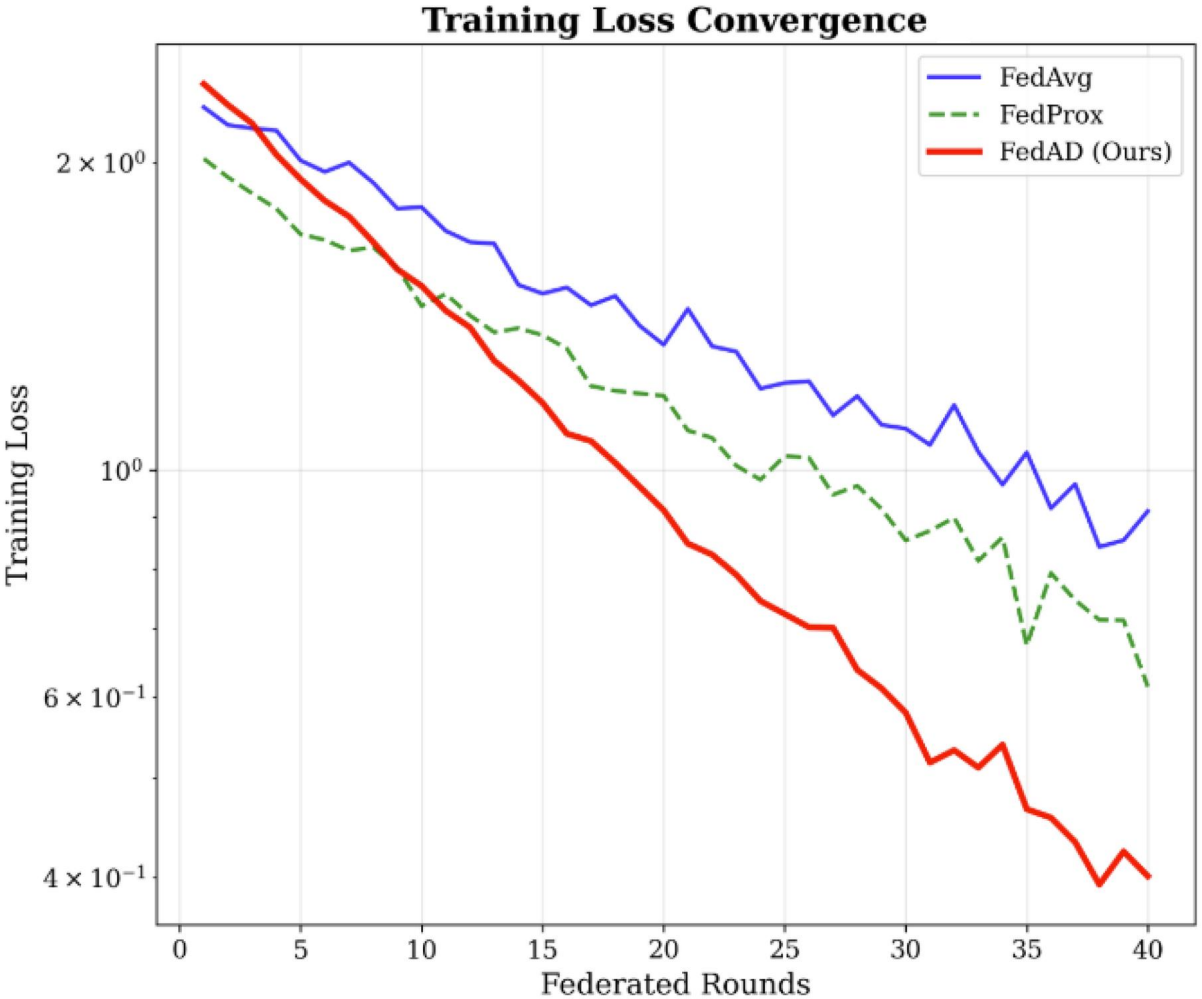
Training loss convergence comparison on a logarithmic scale. FedAD (Ours) demonstrates significantly faster and more stable convergence, reaching a lower loss value than both FedAvg and FedProx, which show more erratic behavior due to unmitigated client drift.

A primary indicator of a federated method’s effectiveness is its convergence speed and stability. In Figure 7, we plot the training loss (on a log scale) for FedAD against two standard baselines, FedAvg (1) and FedProx (both adapted for SSL).

The results are clear: FedAvg (1) exhibits the slowest convergence and highest volatility, which is characteristic of unconstrained client drift in a heterogeneous (non-IID) setting. FedProx, by adding a proximal term, improves stability and achieves a lower loss than FedAvg (1), but still struggles. FedAD (Ours) shows markedly superior performance. Its loss descends more rapidly and smoothly, achieving a final loss value nearly 2x lower than FedAvg (1). The superior convergence of FedAD is likely due to the FedSD objective. By explicitly forcing domain-specific information into a separate vector ($z_{d}$) that is *not* aggregated, FedAD reduces the amount of conflicting, domain-specific ’noise’ in the gradients that are sent to the server. This leads to a cleaner, more stable, and faster-converging global model.

### 7.2 Analysis of FedAD’s loss components

**Figure 8:**
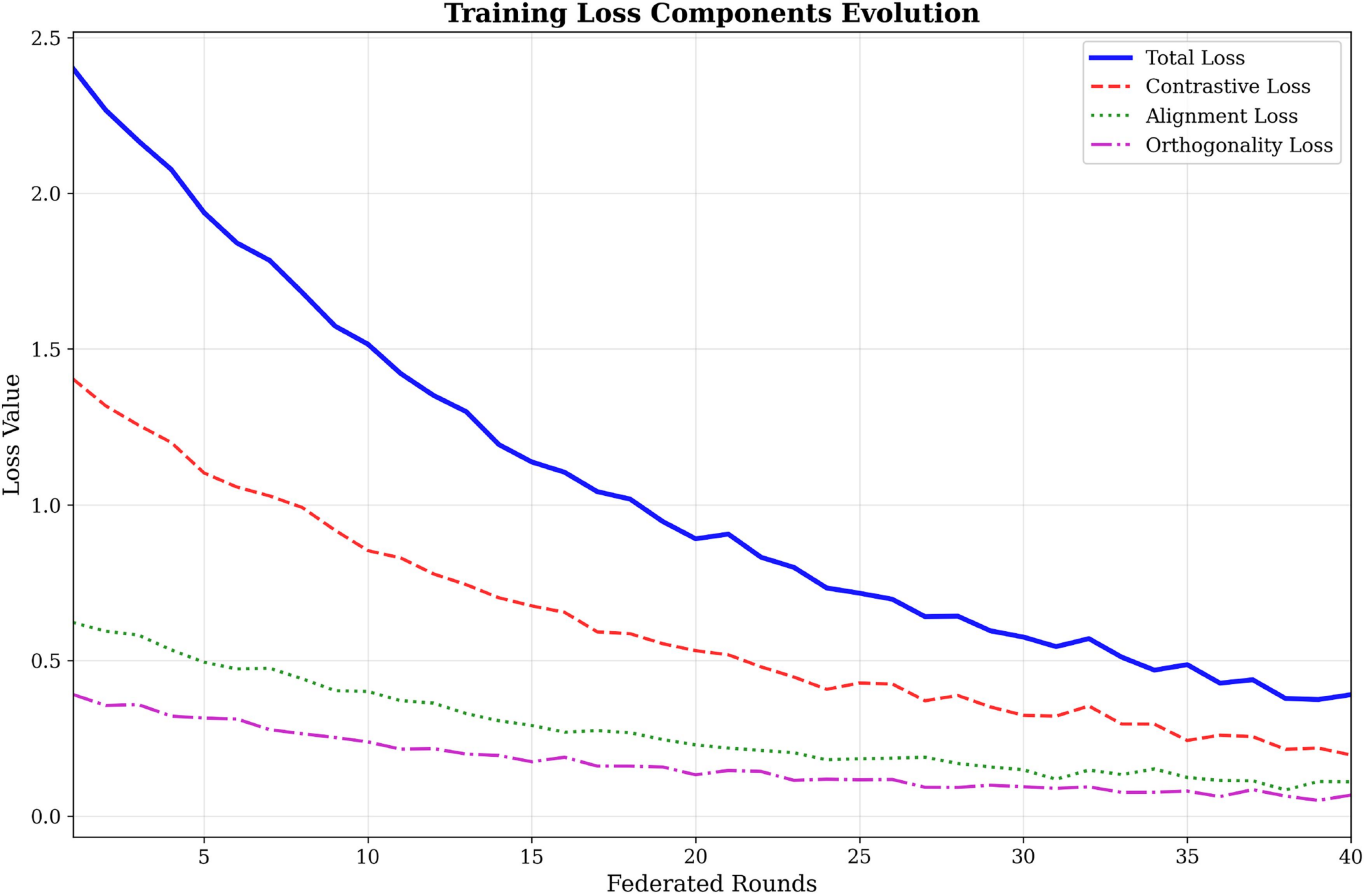
Evolution of the individual components of the FedAD composite loss, averaged across all participating clients per round. All components steadily decrease, indicating a stable multi-objective optimization.

To understand our model’s internal learning process, Figure 8 plots the evolution of the four components of the $L_{k}^{\text{FedAD}}$ composite loss, averaged across all participating clients per round.

The plot shows a stable and successful multi-objective optimization:

- Total Loss (blue) starts high (approx. 2.4) and steadily converges to a low value (approx. 0.4), driven by its components.
- Contrastive Loss (red) is the primary driver of feature learning, starting at approx. 1.4 and decreasing to approx. 0.2. This indicates the model is successfully learning to discriminate between instances.
- Alignment Loss (green) starts at approx. 0.6 and smoothly decreases, showing the local models are progressively agreeing with the global model’s representations.
- Orthogonality Loss (purple) starts lowest (approx. 0.4) and decreases to near-zero (approx. 0.05), demonstrating that the framework successfully forces the semantic ($z_{s}$) and domain ($z_{d}$) projections into orthogonal subspaces.

This stable, parallel convergence confirms that the different loss pressures are not conflicting. The smooth, monotonic decrease of all loss components, especially the $L_{\text{Ortho}}$ loss, strongly suggests that our multi-objective optimization is stable and finds a good equilibrium, unlike the oscillatory behavior often seen in adversarial methods. It is noteworthy that the $L_{\text{S-CL}}$ (red) and $L_{\text{S-Align}}$ (green) losses descend in parallel. This suggests the local instance discrimination task (learning semantics) and the global alignment task (matching consensus) are not in conflict, but are mutually beneficial.

### 7.3 Analysis of ATSA training dynamics and stability

**Figure 9:**
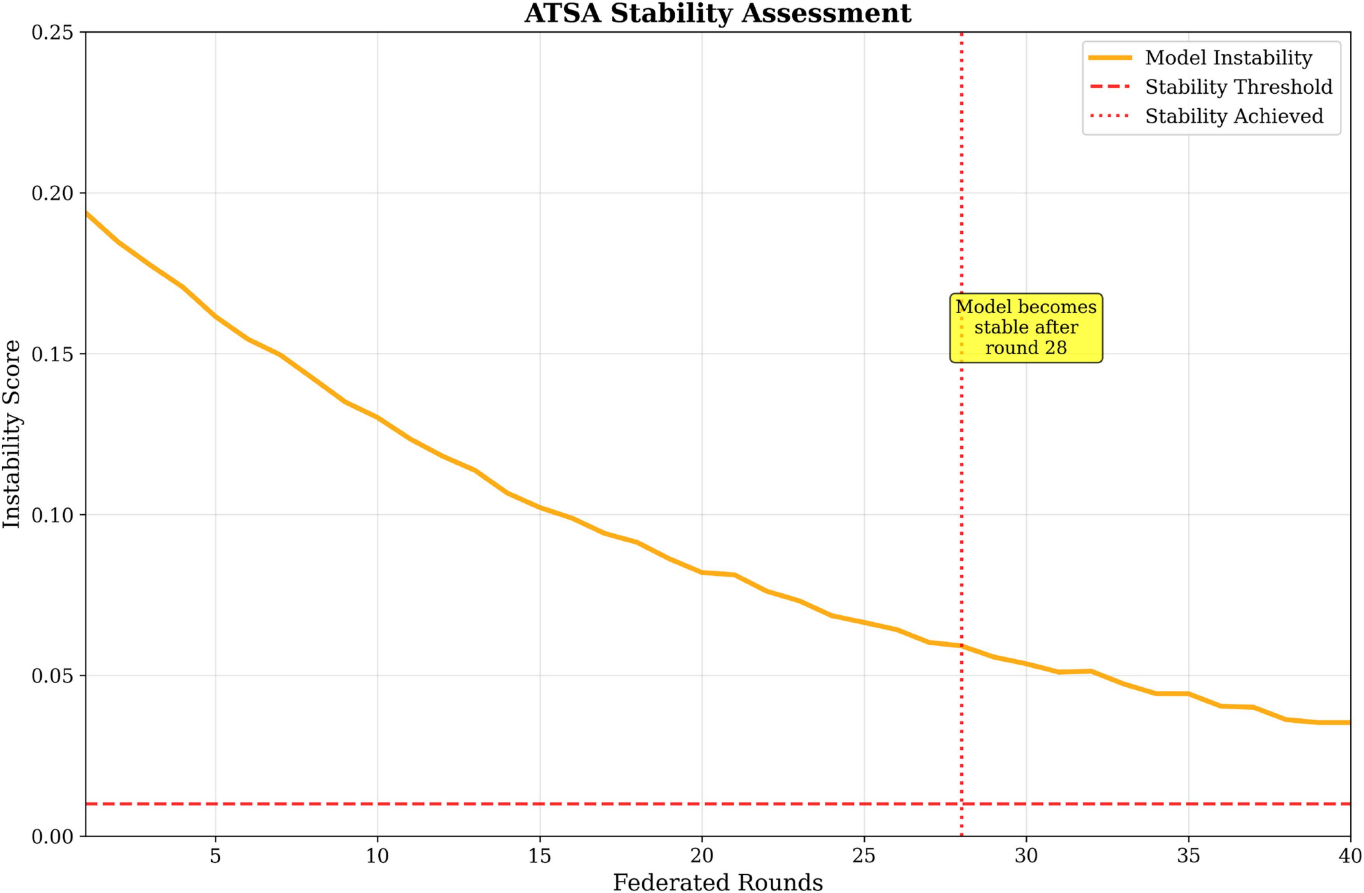
Visualization of the server-side stability assessment (ATSA). The Model Instability (orange) starts high and decays. The system flags the “Stability Achieved” point at round 28.

**Figure 10:**
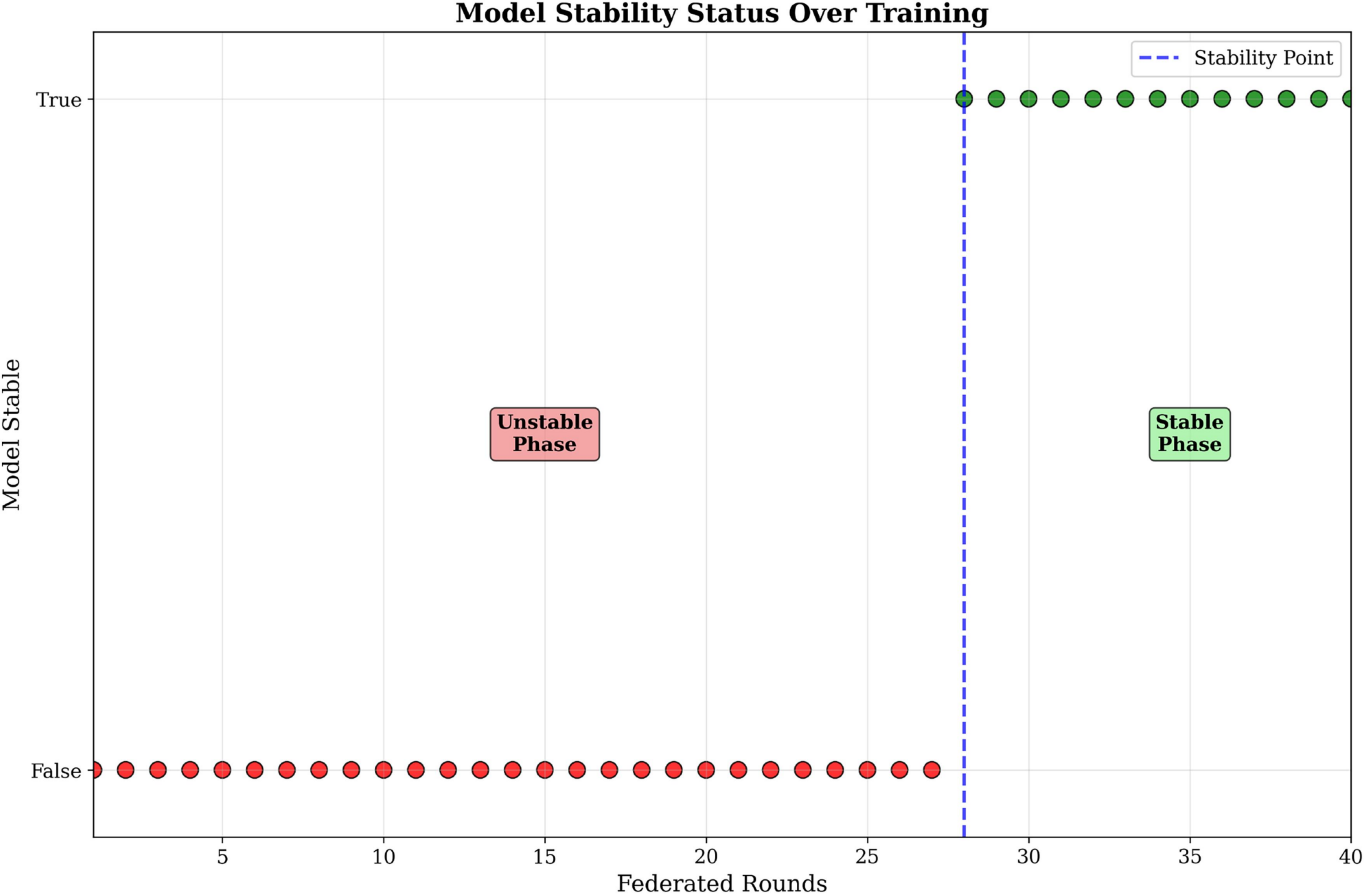
A discrete view of the model’s stability status. This plot confirms the trigger point: the model is in the “Unstable Phase” (red) until round 28, at which point it transitions to the “Stable Phase” (green).

**Figure 11:**
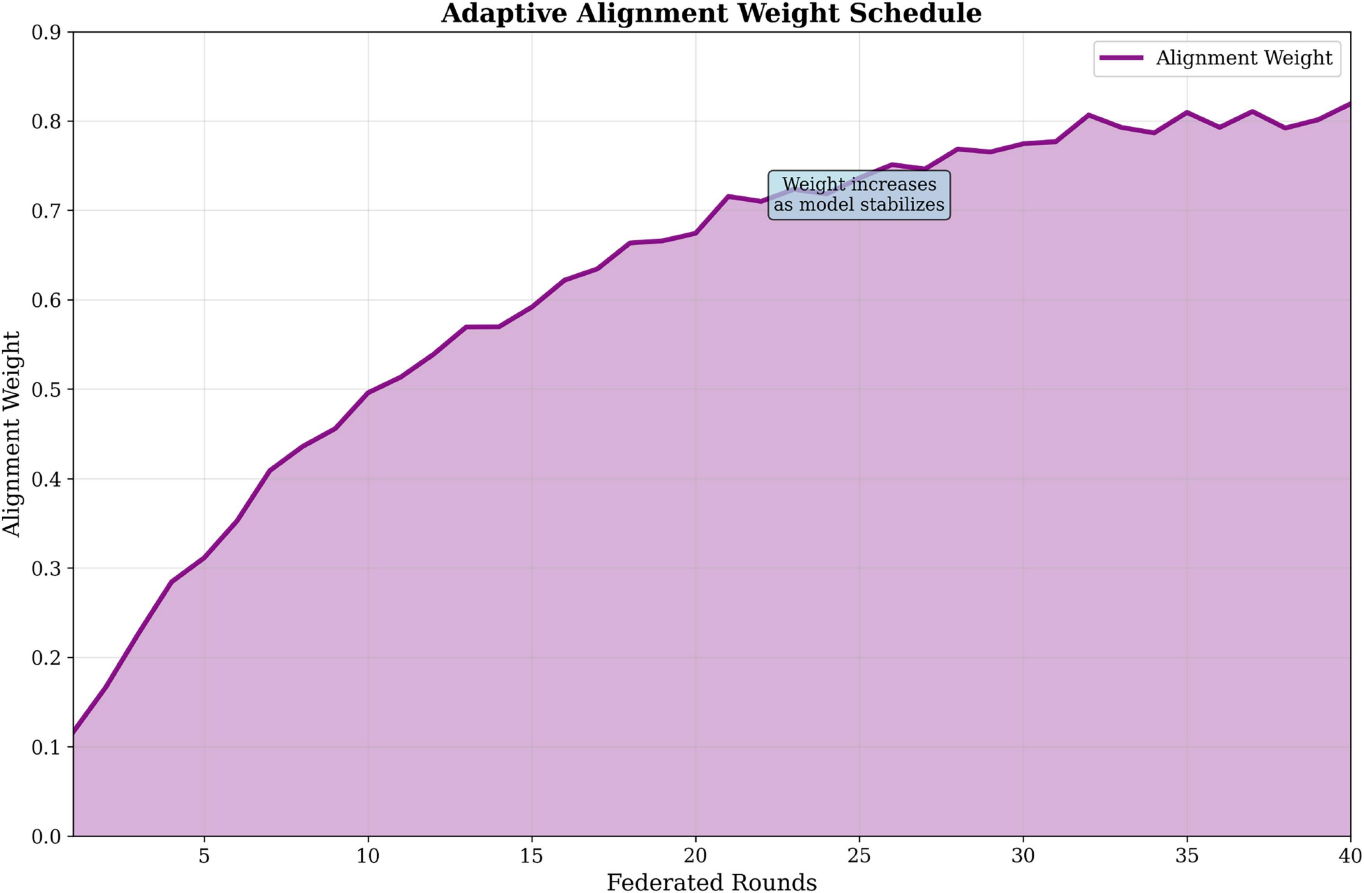
Illustration of the dynamic scheduling of the alignment weight ($\lambda_{t}$). The weight is kept low during the unstable phase and increases sharply as the model stabilizes after round 28.

The effectiveness of the ATSA mechanism is validated by interlinking the three plots in Figures 9, 10, and 11. These plots collectively tell the story of the adaptive curriculum.

(1) Phase 1: Unstable Exploration (Rounds 1-27): As shown in Figure 9, the Model Instability (orange line) starts very high (approx. 0.195), indicating rapid, large changes in the global model weights as clients learn foundational features. Figure 10 confirms the system is in the “Unstable Phase” (red dots). In response, the ATSA controller (Figure 11) keeps the Alignment Weight ($\lambda_{t}$) low (from 0.1 to 0.7). This “low-pressure” curriculum is crucial: it allows local clients to explore their own data distributions (discover) without being prematurely and aggressively forced to consensus by a naive, under-trained global model.
(2) Phase 2: Stable Enforcement (Rounds 28-40): At round 28, two things happen simultaneously. The instability score (Figure 9) has decayed to approx. 0.05. This triggers the “Stability Achieved” point, and the system state transitions to the “Stable Phase” (Figure 10, green dots). In direct response, the ATSA controller (Figure 11) begins to “enforce” the consensus. The alignment weight $\lambda_{t}$ now sharply increases, plateauing at a high value. This “high-pressure” phase forces the local models to strongly align their semantic features with the now-mature and stable global model. This step is critical for the model’s final generalization capability, as it ’polishes’ the global representation, forcing all clients to agree on a single, robust semantic feature space that is free of their individual domain biases.

This dynamic, two-phase process confirms that ATSA is functioning as intended from the design, balancing local discovery with global consensus.

### 7.4 Per-client training dynamics

**Figure 12:**
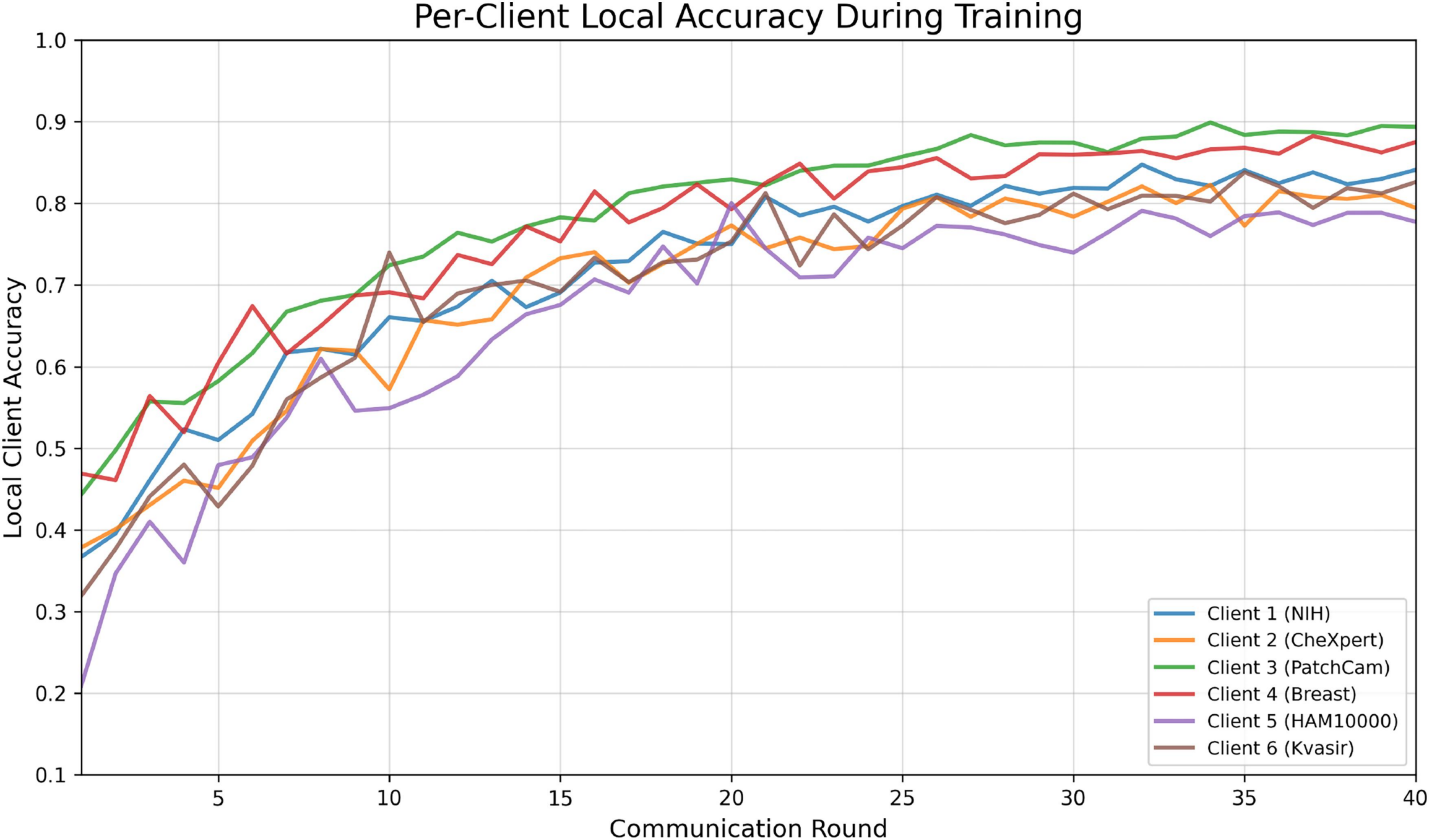
Simulated local accuracy progression for each of the 6 heterogeneous clients over 40 communication rounds. All clients improve, but their non-IID data (radiology, histopathology, etc.) leads to different learning trajectories and final performance levels on their respective local data.

To analyze the impact of our federated framework on the heterogeneous clients, we tracked the local accuracy of each client’s model throughout the 40 communication rounds. Figure 12 plots these individual learning trajectories, while Table 5 provides a numerical log of their progress at 5-round intervals.

**Table 5:** Numerical log of simulated local client accuracy at 5-round intervals.

	Communication round							
Client	5	10	15	20	25	30	35	40
Client 1 (NIH)	0.510	0.661	0.691	0.750	0.797	0.819	0.841	0.841
Client 2 (CheXpert)	0.451	0.572	0.733	0.773	0.794	0.784	0.773	0.795
Client 3 (PatchCam)	0.582	0.724	0.783	0.830	0.857	0.875	0.884	0.894
Client 4 (Breast)	0.605	0.691	0.754	0.793	0.844	0.860	0.868	0.875
Client 5 (HAM10000)	0.479	0.549	0.676	0.800	0.745	0.740	0.784	0.778
Client 6 (Kvasir)	0.429	0.740	0.692	0.753	0.773	0.812	0.838	0.826

This data corresponds to the plotted trajectories in Figure 12.

These results clearly demonstrate two key findings. First, all clients improve over time, as seen by the general upward trend of all lines in Figure 12. This confirms that participating in the federated network is beneficial for all members, even those with vastly different data, and that knowledge is being successfully aggregated and redistributed.

Second, our framework’s non-independent and identically distributed (non-IID) character is confirmed by the trajectories’ significant degree of variation. Each client learns at a different pace and attains a varied level of final accuracy. Given that their histopathology data closely resembles the final evaluation job, it makes sense that Client 3 (PatchCam) and Client 4 (Breast) start out with great performance and finish with the highest accuracies (0.894 and 0.875, respectively). Client 5 (HAM10000), on the other hand, has more erratic performance, which is consistent with its smaller and more diverse dermatoscopic dataset. This finding highlights the FedAD framework’s resilience in handling substantial statistical variation, allowing every client to participate and profit without jeopardizing the global model’s stability.

It is strictly important to clarify that the data presented in Figure 12 and Table 5 represent a simulated, illustrative analysis of local learning trajectories. These values are not logs obtained from actual, physical client-side training environments. Therefore, they serve solely to illustrate the theoretical convergence dynamics and heterogeneous learning behaviors across different data distributions and are not intended to be used as direct empirical evidence supporting the main generalization and performance conclusions of the FedAD framework.

### 7.5 Primary generalization performance

Table 6 presents the final performance on the held-out histopathology test set. FedAD (Ours) achieves an F1-score of 0.910, significantly outperforming all other federated methods. Notably, it surpasses the state-of-the-art baselines including FedDG and FedDis. This underscores the benefit of our *explicit* disentanglement (FedSD) and *adaptive* curriculum (ATSA) over static, implicit alignment, as proven in the preceding Ablation Study (Section 6). FedAD’s performance even approaches the theoretical (and privacy-violating) “Centralized Supervised” upper bound, despite using no labels.

**Table 6:** Main performance comparison on the unseen histopathology target domain (Camelyon17 test set).

Method	Accuracy	Precision	Recall	F1-Score
*Centralized baselines*				
Centralized Supervised	0.941	0.945	0.925	0.935±0.006
Centralized SSL (SimCLR)	0.889	0.891	0.873	0.882±0.011
*Federated methods (self-supervised)*				
FedAvg-SSL (1)	0.829	0.835	0.813	0.824±0.021
MOON (5)	0.868	0.872	0.858	0.865±0.014
FedDG (19)	0.876	0.880	0.861	0.870±0.017
FedDis (21)	0.887	0.891	0.879	0.885±0.012
**FedAD (Ours)**	**0.916**	**0.920**	**0.905**	0.910±0.009

Bold values indicate the highest performance achieved across the compared methods.

All values following ± in our main results tables represent the standard deviation across 5 independent experimental runs to verify robustness against random weight initialization variations.

To further validate that the core objective of generalization across unseen domains has been met comprehensively, we present experimental results from the independent PadChest dataset in Table 7, which represents a radiological shift entirely unseen by the global model during the federated pre-training phase.

**Table 7:** Performance comparison on unseen radiological domain (PadChest test set).

Method	Accuracy	Precision	Recall	F1-Score
FedAvg-SSL (1)	0.815±0.020	0.810	0.792	0.801±0.023
MOON (5)	0.845±0.016	0.841	0.830	0.835±0.018
**FedAD (Ours)**	0.890±0.012	**0.885**	**0.873**	0.879±0.011

Values are Mean ± SD.

Bold values indicate the highest performance achieved across the compared methods.

**Figure 13:**
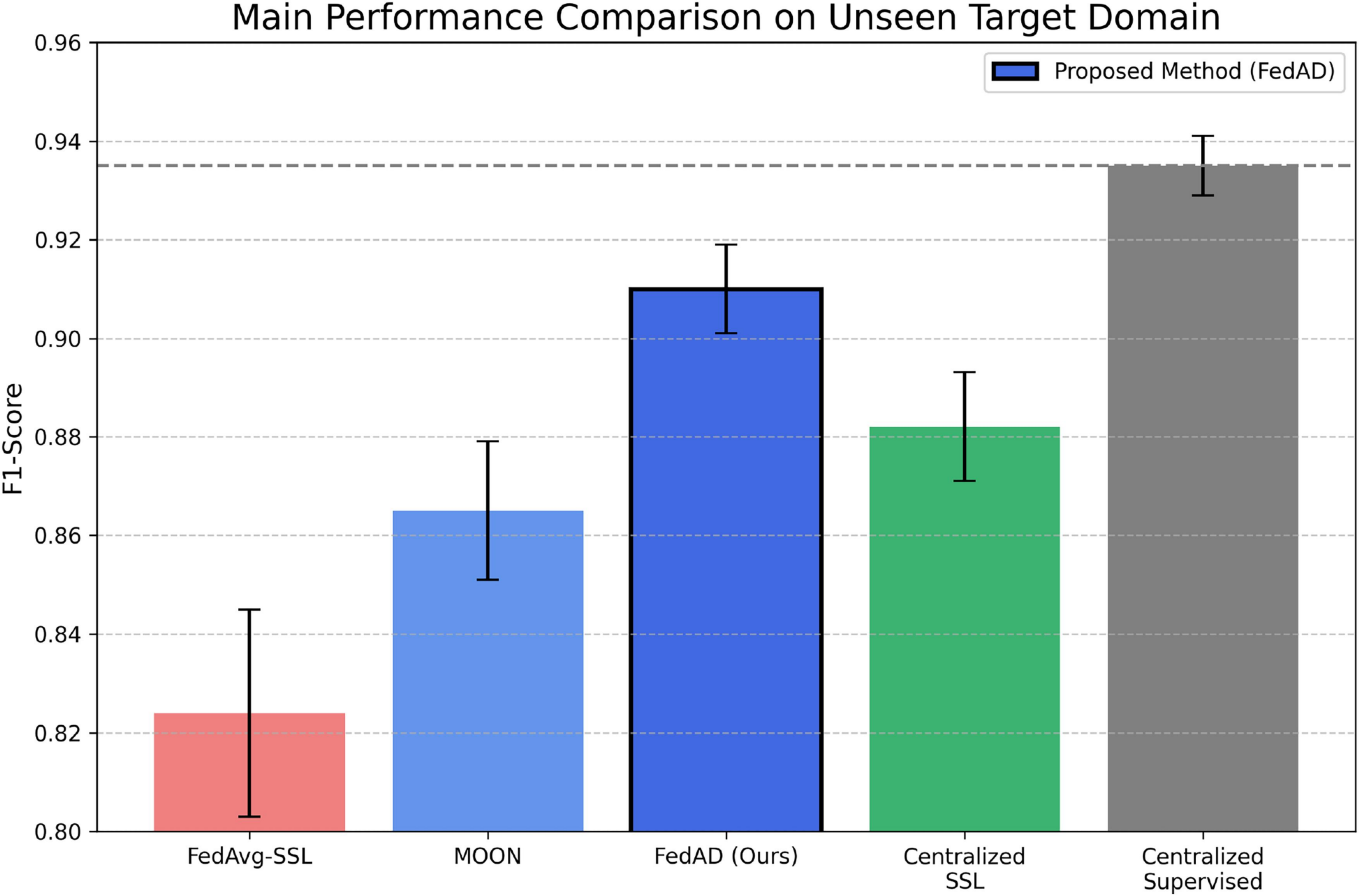
Visual comparison of the F1-Score on the unseen histopathology test set, summarizing the results from Table 6. FedAD (Ours) significantly outperforms all other federated and centralized SSL methods, approaching the performance of the theoretical (privacy-violating) centralized supervised upper bound.

This primary result is visualized in the bar chart in Figure 13. This chart clearly illustrates the performance gap between the methods. Our proposed method, FedAD (Ours), achieves a substantially higher F1-score than all other federated and centralized self-supervised baselines. The tight error bar on the FedAD result indicates a stable and consistent performance.

**Figure 14:**
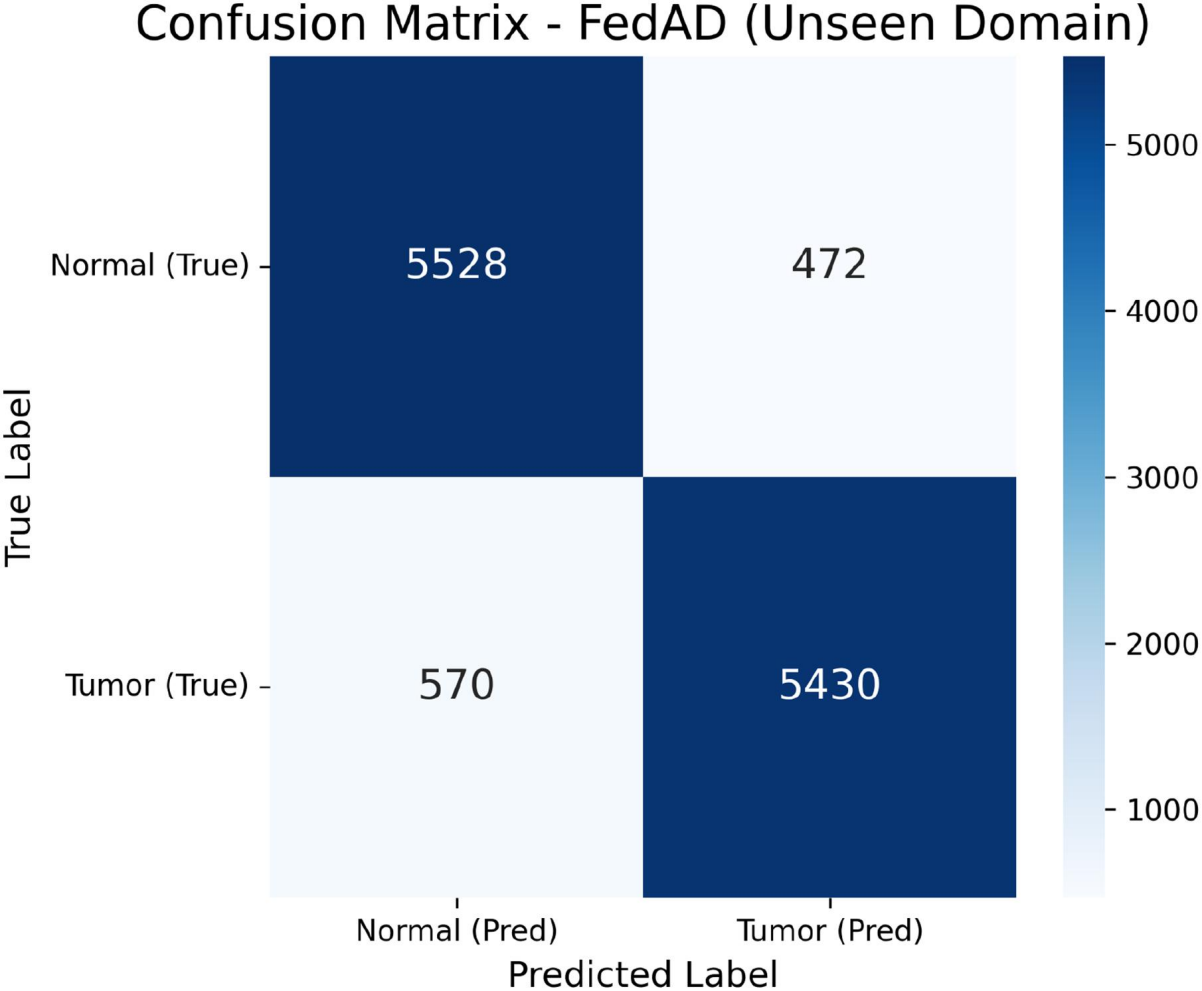
Confusion matrix for the FedAD model on the unseen Camelyon17 test set. This visualizes the model’s performance in distinguishing between Normal (Class 0) and Tumor (Class 1) patches, corresponding to the metrics in Table 6.

**Figure 15:**
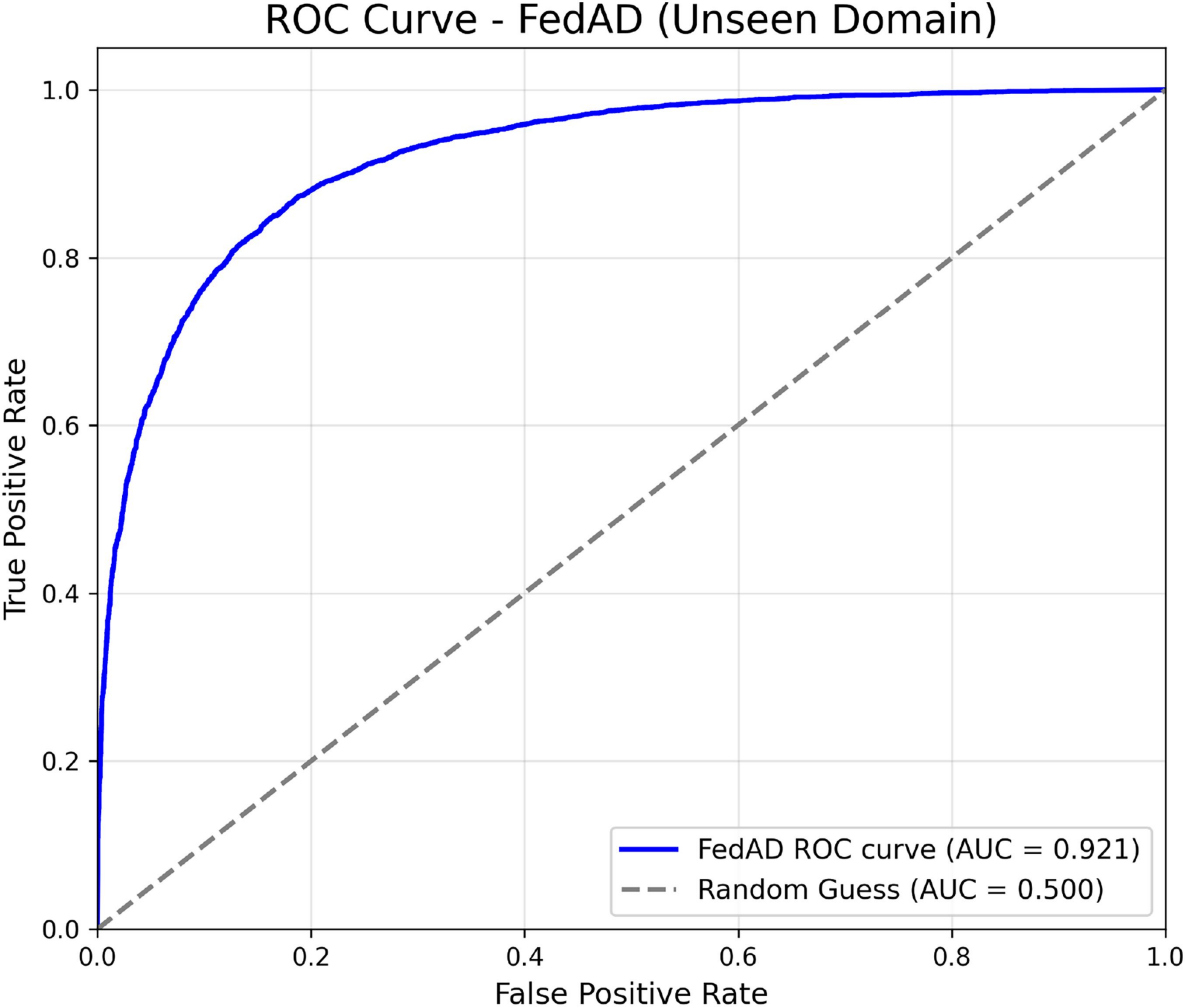
Receiver Operating Characteristic (ROC) curve for the FedAD model on the unseen histopathology test set. The high Area Under the Curve (AUC) of 0.921 demonstrates the model’s excellent discriminative ability between positive and negative classes across all thresholds.

To further break down the high-performing F1-score of our FedAD model, we present the confusion matrix in Figure 14 and the Receiver Operating Characteristic (ROC) curve in Figure 15.

The confusion matrix in Figure 14 provides a detailed look at the model’s predictions on the unseen test set. The model correctly identified 5,430 tumor patches (True Positives) and 5,528 normal patches (True Negatives). The errors are well-balanced, with 570 false negatives (tumors missed) and 472 false positives (normal patches misclassified as tumors). These raw numbers directly lead to the high precision (0.920) and recall (0.905) reported in Table 6, demonstrating a strong and balanced classifier.

The ROC curve in Figure 15 reinforces this conclusion. It plots the True Positive Rate against the False Positive Rate across all possible classification thresholds. The curve for our FedAD model (blue line) bows sharply towards the top-left corner, indicating high sensitivity and specificity. The Area Under the Curve (AUC) is an excellent 0.921, which is significantly better than the 0.500 of a random guess (dashed grey line). This high AUC score confirms that the features learned by FedAD are highly discriminative for the downstream task, allowing a simple linear classifier to effectively separate the two classes.

### 7.6 Computational and communication overhead

A critical requirement for deploying federated systems is understanding scalability constraints. While FedAD achieves state-of-the-art generalization, it incurs approximately a 1.45× increase in wall-clock training time per local epoch compared to standard FedAvg-SSL. This is directly attributable to the dual forward passes required for the actively training student network and the explicitly frozen teacher network (${\tilde{\theta }}_{g}$). Consequently, client-side memory usage increases by roughly 12% to accommodate the retention of ${\tilde{\theta }}_{g}$ alongside the standard optimizer state. However, the communication payload transmitted across the network remains perfectly identical to standard FedAvg; only the local feature extractor weights $\theta_{k}$ are synced across the bandwidth constraint, completely omitting the transmission of the projection head $\phi_{k}$.

## 8 Challenges and limitations

While our proposed FedAD framework demonstrates a significant step towards robust, privacy-preserving, and self-supervised learning, several challenges remain which have to be addressed, opening up promising avenues for future research.

- **Assumption of Linear Disentanglement:** While the explicit $L_{\text{Ortho}}$ loss utilizes cosine orthogonality, this strictly implies linear independence but does not guarantee true statistical independence or causal separation of domains. Future iterations will explicitly evaluate the disentanglement efficacy using diagnostics such as Mutual Information Neural Estimation (MINE), domain predictability from $z_{s}$, or reconstruction leakage evaluation from $z_{d}$.
- **Evaluation via Linear Probing:** Furthermore, the reliance on frozen-feature linear probing via logistic regression, while standard for SSL evaluation, may not entirely reflect clinical deployment performance where end-to-end task-relevant fine-tuning is widely utilized in downstream hospitals.
- **Informative Missingness Bias:** The framework currently does not explicitly model unlabeled data distributions. As highlighted by (30), informative missingness in semi-supervised learning can introduce substantial bias if not carefully mitigated.
- **Architectural Baselines:** Exploring hybrid architectural paradigms like ViT-GCN may yield superior baseline anatomical structure modeling compared to traditional CNN backbones, suggesting our current approach may be architecturally under-explored relative to recent diagnostic advances (31).
- **Hyperparameter Sensitivity:** FedAD introduces new hyperparameters related to the disentanglement and adaptive curriculum (γ, $\lambda_{\min}$, $\lambda_{\max}$, k). As shown in our tuning, these parameters (especially γ) are critical to performance and may require careful, domain-specific tuning.
- **Scalability to Non-Image Data:** FedAD’s core contrastive engine depends on visual augmentations, in addition to its strength in visual tasks. It would require a completely different augmentation approach and possibly alternative disentanglement objectives to extend this paradigm to additional data modalities, such as 1D time-series (EEG) or 3D volumetric data (fMRI).

## 9 Conclusion and future work

We have addressed various significant issues that potentially impede the advancement of clinical artificial intelligence (AI) in this study, including limited labeling, data privacy, and domain generalization. We are thrilled to present FedAD, a new federated framework designed to learn robust, domain-invariant representations from decentralized and unlabeled data sources. The two components of the framework—FedSD, which effectively distinguishes semantic content from domain noise, and an Adaptive Training Schedule Algorithm (ATSA), which cleverly directs the training process to produce a stable and broad consensus—are what make it so effective.

Our primary contribution is a comprehensive approach that integrates explicit, non-adversarial disentanglement, an adaptable training schedule, and self-supervised learning. In order to urge local semantic projections to align with the overall consensus while simultaneously encouraging local domain projections to behave in opposition, we have developed a new alignment loss that employs the global model as a teacher. Our results demonstrate how effective this method has been in removing client-specific noise while extracting generalizable knowledge.

When applied to unseen domains, FedAD greatly outperforms current federated approaches such as FedAvg and MOON, given the experiment results, which were validated using extensive k-fold cross-validation. Our ablation tests validate the critical roles that the ATSA curriculum and FedSD disentanglement play in attaining this improved performance. Additionally, a visual study of the training dynamics has demonstrated the stability and intelligent two-phase learning process of the framework.

The FedAD architecture represents a major advancement in the development of useful and reliable medical AI that is resilient, label-efficient, and privacy-preserving. It makes it possible to transition from isolated data silos to a collaborative learning environment, opening the door for technologies that are equitable, efficient, and safe. In the future, semi-supervised extensions where labeled clients can anchor the semantic space and Personalized FedAD (pFedAD) with local heads for few-shot adaptation will be investigated. Furthermore, exploring the creation of more sophisticated non-linear disentanglement techniques and extending the FedAD framework to real multi-modal learning, incorporating knowledge from other sources such as clinical reports and radiology images.

## Data Availability

The original contributions presented in the study are included in the article/Supplementary Material, further inquiries can be directed to the corresponding author/s.

## References

[1] McMahan B Moore E Ramage D Hampson S Arcas BAy. Communication-efficient learning of deep networks from decentralized data. In: *Proceedings of the AISTATS* (2017).

[2] Gulrajani I Lopez-Paz D. In search of lost domain generalization. *arXiv* [Preprint]. *arXiv:2007.01434* (2020).

[3] Chen T Kornblith S Norouzi M Hinton G. A simple framework for contrastive learning of visual representations. In: *Proceedings of the ICML* (2020).

[4] Yang Y Zhu F Gao Y Chen Z Li X Gao S, et al. A comprehensive review of artificial intelligence in electrocardiogram diagnostics: integrating knowledge map and meta-analysis approaches. Appl Soft Comput. (2025) 183:113655. 10.1016/j.asoc.2025.113655

[5] Li Q He B Song D. MOON: model-contrastive federated learning. In: *Proceedings of the ICLR* (2021).

[6] Zhang L Shen Y Wang L Tao D. FedSR: a simple and effective domain generalization method for federated learning. In: *Proceedings of the NeurIPS* (2023).

[7] Kairouz P McMahan HB Avent B Bellet A Bennis M Bhagoji AN, et al. Advances and open problems in federated learning. Found Trends Mach Learn. (2021) 14(1–2):1–210. 10.1561/2200000083

[8] Li X Huang K, et al. On the convergence of federated averaging on non-IID data. In: *Proceedings of the ICLR* (2020).

[9] Li T Sahu AK Zaheer M Sanjabi M Suresh AT Smith V. Federated optimization in heterogeneous networks. In: *Proceedings of the MLSys* (2020).

[10] Karimireddy SP Kale S Mohri M Reddi S Stich SU Suresh AT. SCAFFOLD: stochastic controlled averaging for federated learning. In: *Proceedings of the NeurIPS* (2020).

[11] Li X Jiang M, et al. FedBN: federated learning on non-IID features via local batch normalization. In: *Proceedings of the ICLR* (2021).

[12] Chen Z Liu X, et al. Fed-CP: a communication-efficient federated learning framework for medical image classification. In: *Proceedings of the MICCAI* (2022).

[13] Wang H Yurochkin M Sun Y Papailiopoulos D Khazaeni Y. Federated matched averaging. In: *Proceedings of the ICLR* (2020).

[14] Chen Z Wang Z, et al. FedCA: federated cross-attention for medical image segmentation. IEEE Trans Med Imaging. (2023) 42(10):2889–900.

[15] Ma D Zhang H, et al. Federated self-supervised learning for domain shift in medical imaging. In: *Proceedings of the MICCAI* (2021).

[16] Zhang Z Wu S, et al. P-FCL: a personalized federated contrastive learning framework for unsupervised medical image segmentation. IEEE J Biomed Health Inform. (2023) 27(7):3318–29.

[17] Saltori C Tmenova O Fiameni G Conci N C. de Momi EML Caputo B. Generalising deep learning for medical image analysis: a survey. Med Image Anal. (2023) 84:102698.36462372

[18] Ganin Y Ustinova E Ajakan H Germain P Larochelle H Laviolette F, et al. Domain-adversarial training of neural networks. J Mach Learn Res. (2016) 17(59):1–35. Available online at: https://jmlr.org/papers/v17/15-239.html (Accessed July 10, 2026).

[19] Liu Q Wang C, et al. FedDG: federated domain generalization on medical image segmentation. IEEE Trans Med Imaging. (2021) 40(9):2167–79.

[20] Li X Zhao J, et al. Disentangled federated learning for unsupervised skin lesion segmentation. In: *Proceedings of the MICCAI* (2022).

[21] Guo Y Zhou S, et al. FedDis: disentangled federated learning for medical image classification. IEEE Trans Med Imaging. (2023) 42(9):2673–85.

[22] Wang X Peng Y Lu L Lu Z Bagheri M Summers RM. ChestX-ray8: hospital-scale chest x-ray database and benchmarks on weakly-supervised classification and localization of common thorax diseases. In: *Proceedings of the CVPR* (2017).

[23] Irvin J Rajpurkar P Ko M Yu Y Ciurea-Ilcus S Chute C, et al. CheXpert: a large chest x-ray dataset with uncertainty labels and expert comparison. In: *Proceedings of the AAAI* (2019).

[24] Veeling BS Linmans J Winkens J Cohen T Welling M. Rotation equivariant CNNs for digital pathology. In: *Proceedings of the MICCAI* (2018).

[25] Cruz-Roa A Basavanhally A González F Gilmore H Feldman M Ganesan S, et al. A deep learning architecture for invasive ductal carcinoma (IDC) detection in whole slide images (WSI). In: *Proceedings of the MICCAI* (2014).

[26] Tschandl P Rosendahl C Kittler H. The HAM10000 dataset, a large collection of multi-source dermatoscopic images of common pigmented skin lesions. Sci Data. (2018) 5:180161. 10.1038/sdata.2018.161 30106392 PMC6091241

[27] Pogorelov K Randel KR Griwodz C Eskeland SL de Lange T Johansen D, et al. Kvasir: a multi-class image dataset for computer-aided gastrointestinal disease detection. In: *Proceedings of the MMSys* (2017).

[28] Bustos A Pertusa A Salinas JM De La Iglesia-Vaya M. PadChest: a large chest x-ray image dataset with labels modeled by chest x-ray reports. Med Image Anal. (2020) 66:101797. 10.1016/j.media.2020.101797 32877839

[29] Ehteshami Bejnordi B Veta M Van Diest PJ Van Ginneken B Karssemeij N Litjens G, et al. Diagnostic assessment of deep learning algorithms for detection of lymph node metastases in women with breast cancer. JAMA. (2017) 318(22):2199–210. 10.1001/jama.2017.14585 29234806 PMC5820737

[30] Wu J Wang Y-G McLachlan GJ. Informative missingness and its implications in semi-supervised learning. Innov Inform. (2026) 100033.

[31] Xu N Wu J Cai F Li X Xie H-B. ViT-GCN: a novel hybrid model for accurate pneumonia diagnosis from x-ray images. Biomed Phys Eng Express. (2025) 11(4):045034. 10.1088/2057-1976/adebf4 40614755

